# PLCε regulates prostate cancer mitochondrial oxidative metabolism and migration via upregulation of Twist1

**DOI:** 10.1186/s13046-019-1323-8

**Published:** 2019-08-05

**Authors:** Jiaxin Fan, Yanru Fan, Xiao Wang, Lingfang Niu, Limei Duan, Jinxiao Yang, Luo Li, Yingying Gao, Xiaohou Wu, Chunli Luo

**Affiliations:** 1 Key Laboratory of Clinical Laboratory Diagnostics (Ministry of Education), College of Laboratory Medicine, School of Laboratory Medicine, Chongqing Medical University, No.1, Yixueyuan Road, Chongqing, 400016 People’s Republic of China; 2 Department of Urology Surgery, Neijiang First People’s Hospital, Neijiang, People’s Republic of China; 3 Department of Urology, The First Affiliated Hospital of Chongqing Medical University, Chongqing, People’s Republic of China; 4 Key Laboratory of Clinical Laboratory Diagnostics (Ministry of Education), College of Laboratory Medicine, School of Laboratory Medicine, Chongqing Medical University, Chongqing, 400016 People’s Republic of China

**Keywords:** PLCε, Twist1, Prostate cancer, Mitochondrial metabolic, Migration, MAPK, Ubiquitination

## Abstract

**Background:**

Metabolic rewiring is a common feature of many cancer types, including prostate cancer (PCa). Alterations in master genes lead to mitochondrial metabolic rewiring and provide an appealing target to inhibit cancer progression and improve survival. Phospholipase C (PLC)ε is a regulator of tumor generation and progression. However, its role in mitochondrial metabolism remains unclear.

**Methods:**

The GEO, The Cancer Genome Atlas, and the GTEx databases were used to determine Twist1 mRNA levels in tumors and their non-tumor counterparts. Fifty-five PCa and 48 benign prostatic hypertrophy tissue samples were tested for the presence of PLCε and Twist1 immunohistochemically. An association between PLCε and Twist1 was determined by Pearson’s correlation analysis. PLCε was knocked down with a lentiviral short hairpin RNA. Mitochondrial activity was assessed by measuring the oxygen consumption rate. Western blotting analyses were used to measure levels of PPARβ, Twist1, phosphorylated (p)-Twist1, p-MEK, p-ERK, p-P38, and p-c-Jun N-terminal kinase (JNK). Cells were treated with inhibitors of MEK, JNK, and P38 MAPK, and an agonist and inhibitor of peroxisome proliferator activated receptor (PPAR) β, to evaluate which signaling pathways were involved in PLCε-mediated Twist1 expression. The stability of Twist1 was determined after blocking protein synthesis with cycloheximide. Reporter assays utilized E-cadherin or N-cadherin luciferase reporters under depletion of PLCε or Twist1. Transwell assays assessed cell migration. Finally, a nude mouse tumor xenograft assay was conducted to verify the role of PLCε in tumor formation.

**Results:**

Our findings revealed that the expression of PLCε was positively associated with Twist1 in clinical PCa samples. PLCε knockdown promoted mitochondrial oxidative metabolism in PCa cells. Mechanistically, PLCε increased phosphorylation of Twist1 and stabilized the Twist1 protein through MAPK signaling. The transcriptional activity of Twist1, and the Twist1-mediated epithelial-to-mesenchymal transition, cell migration, and transcription regulation, were suppressed by PLCε knockdown and by blocking PPARβ nuclear translocation. The tumor xenograft assay demonstrated that PLCε depletion diminished PCa cell tumorigenesis.

**Conclusions:**

These findings reveal an undiscovered physiological role for PLCε in the suppression of mitochondrial oxidative metabolism that has significant implications for understanding PCa occurrence and migration.

**Electronic supplementary material:**

The online version of this article (10.1186/s13046-019-1323-8) contains supplementary material, which is available to authorized users.

## Background

Prostate cancer (PCa), a leading cause of cancer mortality in males worldwide,is associated with rapid treatment resistance, metastasis, and death [1]. PCa metastasis is the main cause of death [2]. Therefore, defining the molecular basis of metastasisis urgently required within a changing tumor microenvironment.

Cells continually balance their metabolism according to the availability of extracellular nutrients and the evolving microenvironment to meet the needs of their current state and function [3, 4]. Mitochondria, the metabolic cores of cells, play an essential role in this dynamic [3]. Moreover, metabolic rewiring, a common feature of many cancer types, is an appealing target to inhibit cancer progression and improve survival [5].

Numerous reports indicate that the main trigger for metabolic reprogramming is an alteration in cancer genes that remodels the signaling pathways involving one or a few enzymes [6]. Peroxisome proliferator-activated receptor (PPAR) gamma co-activator-1 alpha (PGC-1α) was first identified as a PPARγ-interacting protein in brown fat [7]. Expression of PGC-1α powerfully induces mitochondrial oxidative metabolism and uncoupling proteins, and it can interact with a diverse range of transcription factors to control metabolic functions [8, 9]. Although, mitochondrial functions modulated by PGC-1α positively impact the in-vivo growth of breast cancer cell lines [10], human tumor transcriptional data, cell culture, and in-vivo mouse experiments revealed that a PGC1α-modulated transcriptional program promotes oxidative metabolism and a universal catabolic state to restrain PCa growth and metastasis [11]. Another report also demonstrated low oxygen consumption of prostatic tissue in general [12]. Overall, investigations of mitochondrial metabolism in PCa are increasing.

Phospholipase C (PLC)ε, belongings to the phosphoinositide-specific PLC family, is a multifunctional signaling protein and combines both PLC and guanine nucleotide exchange factor activities [13, 14]. PLC isozymes catalyze the cleavage of polyphosphoinositides, such as phosphatidylinositol 4,5-bisphosphate, into two intracellular second messengers, diacylglycerol and inositol 1,4,5-trisphosphate, that regulate protein kinase C activity and calcium mobilization, respectively [15]. Importantly, specific to the Ras-associating and CDC25A domains, PLCε leads to Rap1phosphorylation and B-raf activation, causing an enzyme-induced cascade reaction of mitogen-activated protein kinases (MAPKs) [16].

Even several studies that analyzed human colorectal tumors showed markedly reduced levels of PLCε [17–19]. An increasing number of studies have demonstrated that PLCε is involved in the generation and progression of tumors [20–22], and a single nucleotide polymorphism of esophageal squamous cell carcinoma in the PLCε gene indicates that it potentially serves as an oncogene leading to cellular transformation [23]. However, the functions of PLCε in mitochondrial metabolismin cancer are not yet fully established.

Twist1, a highly conserved transcription factor that contains a basic helix-loop-helixdomain was initially recognized as a mesoderm-inducing factor in Drosophila [24], and it is well-known as a major inducer of epithelial-to-mesenchymal transition (EMT) [25]. Aberrant Twist1 overexpression facilitates EMT, cell motility, and invasive activity, and enhances some features of cancer stem cells via control of downstream gene expression [26, 27]. Recent studies have highlighted that the increase of Twist1 expression in patients is associated with the progression of several human malignancies and poor survival [25, 28, 29].

Accumulating evidence demonstrates that Twist1 exerts multiple activities during carcinogenesis [30]. Notably, Pan et al.found that the nuclear receptor PPARβ mediates the actions of PGC-1α and suppresses mitochondrial metabolism and uncoupling via Twist1. This revealed an unexpected physiological role of Twist1in the maintenance of energy homeostasis and control of metabolism [31]. Furthermore, to our knowledge, the exact mechanisms of Twist1 in modulating tumor metabolism remain elusive.

In this study, we investigated the role of PLCε in PCa metabolic control and attempted to find potential connections between PLCε and Twist1. Our hypothesis was that PLCε knockdown would accelerate PGC-1α-mediated mitochondrial metabolism by modulating Twist1.

## Methods

### Patient and specimens

A total of 48 cases of benign prostatic hypertrophy (BPH) samples and 55 cases of PCa samples were collected from patients at Department of Urinary Surgery, the First Affiliated Hospital of Chongqing Medical University, between 2015 and 2017. All of the tissue samples were diagnosed to be PCa or BPH by histological identification and stored in liquid nitrogen before the experiments. Meanwhile another 97 patient serum samples were collected between 2017 and 2018. All patients shared a similar socioeconomic status and provided informed consent.

### Immunohistochemistry

The tissue specimens were embedded in paraffin overnight and were then sliced into 5-mm-thick sections. Immunohistochemical (IHC) analysis of prostate tissues were performed using antibodies against PLCε (santa cruz) and Twist1 (Absin). The protocols are the same as we published before [32].

### Cells culture

Human prostate cancer cells (PC3, DU145, LNCap) are purchased by two agent companies from the Cell Resource Center of Shanghai Academy of Life Sciences, Chinese Academy of Sciences. The PC3 and LNCap cells were purchased from Shanghai Zhong Qiao Xin Zhou Biotechnology Company. The DU145 cells were purchased from Shanghai Biowing Applied Biotechnology Company. Bicalutamide-resistant (Bica-R) cells was constructed early in our group [33]. The LNCap cells, one of the androgen-dependent prostate cancer cell strains, were treated with bicalutamide (10 μM) (Selleck Chemicals, Houston, TX, USA), respectively for at least 6 months to generate bicalutamide-resistant cells. Cells were cultured in RPMI-1640 (Gibco, USA) medium supplemented with 10% fetal bovine serum (Gibco, USA), 1% penicilin/streptomycin ((Life Technologies, Carlsbad CA) in a humidified incubator of 5% CO_2_ at 37 °C.

### Plasmid and lentiviral vector

Twist1 (Myc-DDK-tagged) vector was buyed from OriGene Technologies (RC202920). 7TFP CDH1 (E-cadherin) reporter was purchased from Addgene (http://www.addgene.org). Twist1 reporter (S717559) was purchased from Active Motif. the N-cadherin reporter was generated as described previously [34]. The first intron (+ 746 to + 3156 bp) from the human N-cadherin gene was obtained by PCR using primers 5′- AAATTTGAGCTCGGCTCTAGGGGCTGGATT-3′ (forward) and 5′-GGTTGGAGATCTTGTTGTTCGGGCGTGTAA-3′(reverse). The sequences of LV-sh-NC, LV-sh-PLCε#1, LV-sh-PLCε#2, LV-sh-PLCε#3, vetor-sh-NC, vetor-sh-Twist1#1, vetor-sh-Twist2#, vetor-sh-Twist1#3 and vetor-sh-PPARβ#1 (all transcripts from Gene Pharma Company, Shanghai, China) are showed in the following: LV-sh-NC-F, TTCTCCGAACGTGTCACGT; LV-sh-NC-R, AAGAGGCTTGCACAGTGCA. LV-sh-PLCε#1-F, GCAGATATCTGATGCCATTGC; LV-sh-PLCε#1-R, CGTCTATAGACTACGGTCCCG; LV-sh-PLCε#2-F; GCTTCTTAACACGGGACTTGG.LV-sh-PLCε#2-R, CGAAGAATTGTGACCTGAACC. LV-sh-PLCε#3-F, GGTTCTCTCCTAGAAGCAACC; LV-sh-PLCε#3-R, CCAAGAGAGGATCTTCGTTGG. vetor-sh-NC-F, CACCGTTCTCCGAACGTGTCACGTTTCAAGAGAACGTGA CACGTTCGGAGAATTTTTTG; vetor-sh-NC-R, GATCCAAAAAATTCTCCGAACGTGTCACGTTCTCTTGAAACGTGACACGTTCGGAGAAC. vetor-sh-Twist1#1-F, CACCGAAGCTGAGCAAGATTCAGACCTTCAAGAGAGGTCTGAATCTTGCTCAGCTTTTTTTTG; vetor-sh-Twist1#1-R, GATCCAAAAAAAAGCTGAGCAAGATTCAGACCTCTCTTGAAGGTCTGAATCTTGATCAGCTTC.vetor-sh-Twist1#2-F, CCGGTGCTGGACTCCAAGATGGCAAGCTCGAGCTTGCCATCTTGGAGTCCAGCTTTTTG; vetor-sh-Twist1#2-R, AATTCAAAAAGCTGGACTCCAAGATGGCAAGCTCGAGCTTGCCATCTTGGAGTCCAGCA. vetor-sh-Twist1#3-F, CCGGTCCTGAGCAACAGCGAGGAAGACTCGAGTCTTCCTCGCTGTTGCTCAGGTTTTTG; vetor-sh-Twist1#3-R, AATTCAAAAACCTGAGCAACAGCGAGGAAGACTCGAGTCTTCCTCGCTGTTGCTCAGGA. vetor-sh-PPARβ#1, TTTCCAAAAAAGAAGGCCCGCAGCATCCTTCTCTTGAAAGGATGCTGCGGGCCTTCTGGG; vetor-sh-PPARβ#2(sc-36305-V) from Santa Cruz Biotechnology.

### Antibodies, cellular protein extracts and Western blotting

Antibodies used were anti-PLCε, anti-PPARβ (santa cruz); anti-PPARα, anti-PPARγ, anti-Twist1, anti- phosphorylated (p)-Twist1 (Ser68), anti-PGC-1α, anti- CPT1B, anti-ERRα, anti-UCP-1, anti-ACADM, anti-MEKK3, anti-MEK4, anti-MEKK2, anti-ERK1/2, anti-p-MEK (S218/S222), anti-MEK, anti-p-JNK (Thr183/Tyr185), anti-JNK (Abcam); anti-p-ERK1/2 (Thr202/Tyr204), anti-p-P38, anti-P38, anti-B-Raf, N-Cadherin, E-Cadherin, Vimentin (Cell Signaling Technology); anti-Ras, anti-H3(wanlei); β-actin(Immunoway biotechnology); Anti-DDK (FLAG) (ORIGENE). The total protein, cell cytoplasmic protein and nuclear protein extracts and western blot assays were performed with the above antibodies by standard protocols we described before [22].

### Quantitative RT-PCR

Total RNA was isolated from cells using TRIzol regents (invitrogen). Reverse transcription was performed with 1 μg RNA with a reverse transcriptase core kit (Eurogentec). For the real-time PCR analysis, aliquots of double-stranded cDNA were amplified using a SYBR Green PCR Kit (TaKaRa). The sequences of the primers were as follows: PLCε sense, 5′-GGAGAATCCTCGGTAG and anti-sense, 5′-GGTTGTCAGCGTATGTCC; Twist1 sense, 5′-TCCGCGTCCCACTAGCA and anti-sense, 5′-AGTTATCCAGCTCCAGAGTCTCTAGAC; PPARβ sense, 5′-GCCGCCCTACAACGAGATCA and anti-sense, 5′-CCACCAGCAGTCCGTCTTTGT; β-actin sense, 5′-GGGACCTGACTGACTACCTC and anti-sense, 5′-ACGAGACCACCTTCAACTCCAC. All gene expression experiments were repeated at least 3 times.

### Immunofluorescent staining

Cells grown on a sterile cover slip were washed with PBS, fixed in 4% paraformaldehyde, permeabilized with 1% Triton X-100 and subsequently subjected to stain with anti-Twist (Abcam) at 4 °C overnight.

### Measurement of oxygen consumption rates

The oxygen consumption rates (OCR) of cells were detected by an oxygen electrode assay with 10 mM glucose. First, baseline cellular oxygen consumption (basal respiration) was measured. After oligomycin (1 μM), OCR depicts ATP-linked respiration (mitochondrial ATP production) by subtracting the oligomycin rate from baseline cellular OCR. fluoro-carbonyl cyanide phenylhydrazone (FCCP, 0.2 μM) collapses the inner membrane gradient, driving the electron transport chain (ETC) to function at maximal rate, and the maximal respiration is derived by subtracting non-mitochondrial respiration from the FCCP OCR. Finally, antimycin A/rotenone (Rot/AA, 0.5 μM) were added to inhibit ETC function, indicating the non-mitochondrial respiration. The mitochondrial respiratory reserve capacity was calculated by subtracting basal from maximal respiration. Data were calculated and graphs were plotted using Agilent Seahorse Wave Desktop software and report generator, MS Excel and GraphPad Prism. All experiments were performed in triplicates.

### Transwell assay

Transwell assay was were performed as previously described [33].

### Dual luciferase reporter assay

Cells were transfected with 1 μg human E-cadherin and N-cadherin reporter and co-transfected with 1 μg sh-Twist1 vetor. Cells were collected 48 h after transfection and analyze during the dual-luciferase reporter assay system (Promega, Madison, WI, USA). The pRL-SV40 vector that shows constitutive expression of Renilla luciferase was co-transfected as an internal control to correct for differences in transfection. Three independent were performed in triplicate.

### Animal studies

PC3 cells (4 × 10^6^ cells) infected with sh-NC lentiviral, sh-PLCε lentiviral and vetor-sh-Twist1 were suspended in a 1:1 dilution of Matrigel and implanted subcutaneously into 6- to 8-week-old male nude mice (Chongqing Medical University Laboratory Animal Center). The growth of the primary tumour was monitored by external caliper measurement every 2–3 days. Six weeks later, the mice were sacrificed and the tumour tissues were surgically removed, measured, and fixed for histology studies.

### Statistical analysis

Data are presented as mean ± SD. Statistical analysis was performed using a one-way analysis of variance and a Student’s t test (two-tailed) was used to compare two groups with the SPSS (Version 17.0) and GraphPad (Prism 5.0) software programs. Throughout this study: *p < 0.05; **p < 0.01; and *** *p* < 0.001.

## Results

### Prognostic significance of Twist1 and PLCε expression in PCa

**Fig. 1:**
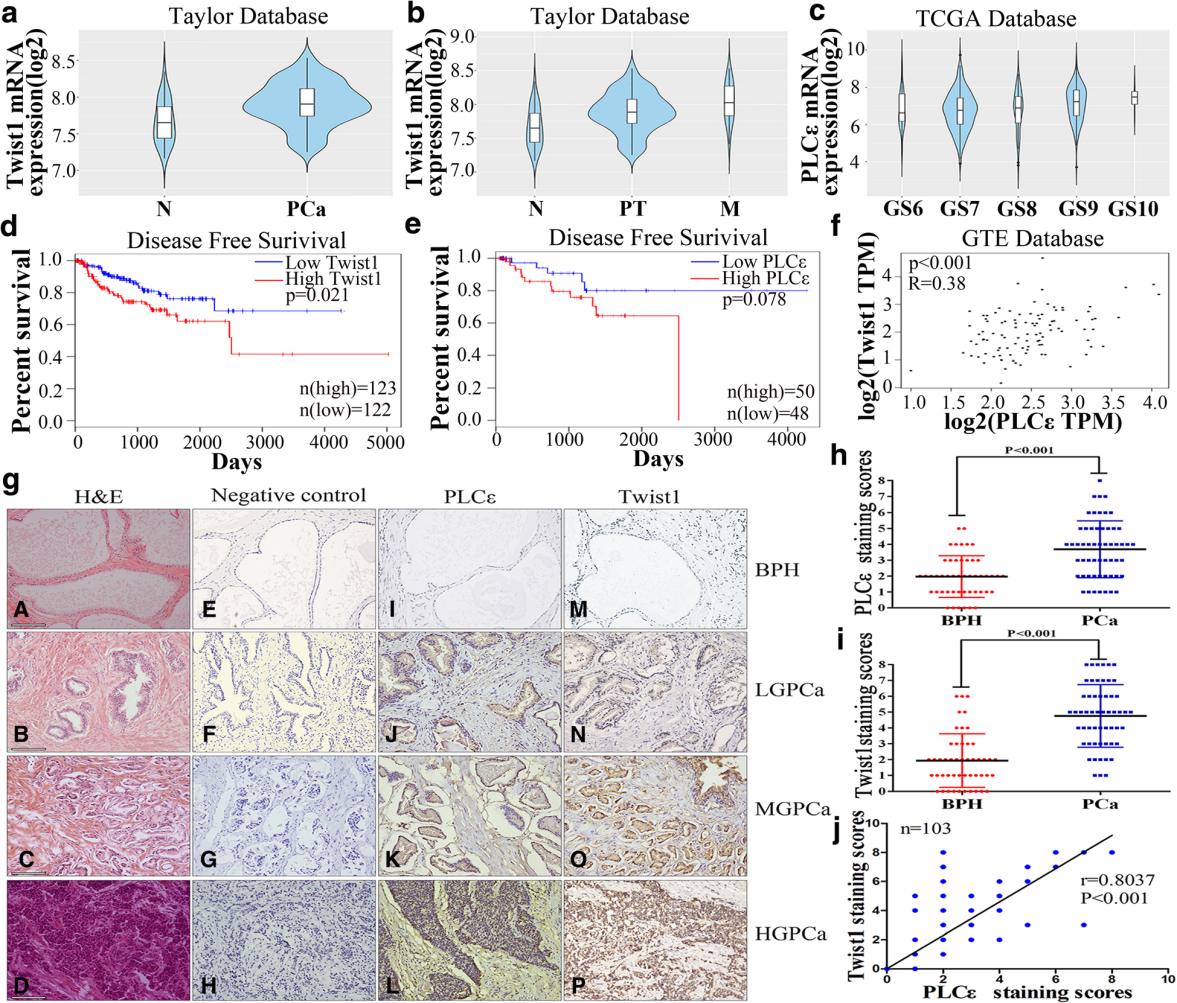
High expression levels of PLCε and Twist1 in PCa tissues. **a** RNA-seq mRNA expression data from the GEO database (GSE21034) was used to compare Twist1 expression between in PCa tumors (PCa) and their non-tumor counterparts (N). * *p* < 0.05. **b** Violin plots depicting the expression of the gene of interest among non-tumoral (N), primary tumor (PT) and metastatic (M) PCa specimens in GEO database. **c** Violin plots depicting the expression of the gene of interest among PCa specimens of the indicated Gleason grade in TCGA Database. **d**, **e** Disease free survival curves of PCa patients according to Twist1 and PLCε mRNA levels. **f** Correlation between the expression of Twist1 and PLCε in PCa patients from GTEx database. **g** Representative haematoxylin and eosin (H&E) staining and IHC staining in 55 PCa tissue samples and 48 BPH samples. Magnification × 400. Bars = 100 μm. H&E staining of prostate tissues were showed (A-D). PBS served as negative control in IHC staining (E-H). Representative IHC staining of different staining intensities used as criteria in staining scoring: none staining was observed in BPH (I, M), mild staining in low-grade (LG) PCa (J, N), moderate staining in middle-grade (MG) PCa (K, 0), and strong staining in high grade (HG) PCa (L, P). **h, i** Average staining scores for PLCε and Twist1 expression in prostate tissues. Data were represented as the means ± SD. **j** The correlation with PLCε and Twist1 in prostate tissues samples was analyzed by Pearson analysis

Analysis of the GEO (GSE21034), The Cancer Genome Atlas (TCGA), and the GTEx databases revealed high levels of Twist1 expression in PCa patients (Fig. 1a and Additional file 1: Figure S1a, *P* < 0.05). The GEO database also showed that Twist1 mRNA levels were significantly higher in metastatic PCa specimens than primary tumors (Fig. 1b, *P* < 0.05). Examining 496 human prostate samples from TCGA showed that the expression of PLCε was significantly associated with the Gleason grade (Fig. 1c, *P* < 0.05). Furthermore, high Twist1 and PLCε mRNA levels were associated with a shorter survival time in PCa patients (Fig. 1d, *P* = 0.021; 1e, *P* = 0.078). Data from GTEx and cBioPortal (http://www.cbioportal.org) revealed a positive correlation between the expression of Twist1 and PLCε (Fig. 1f and Additional file 1: Figure S1b, *P* < 0.001).

**Fig. 2:**
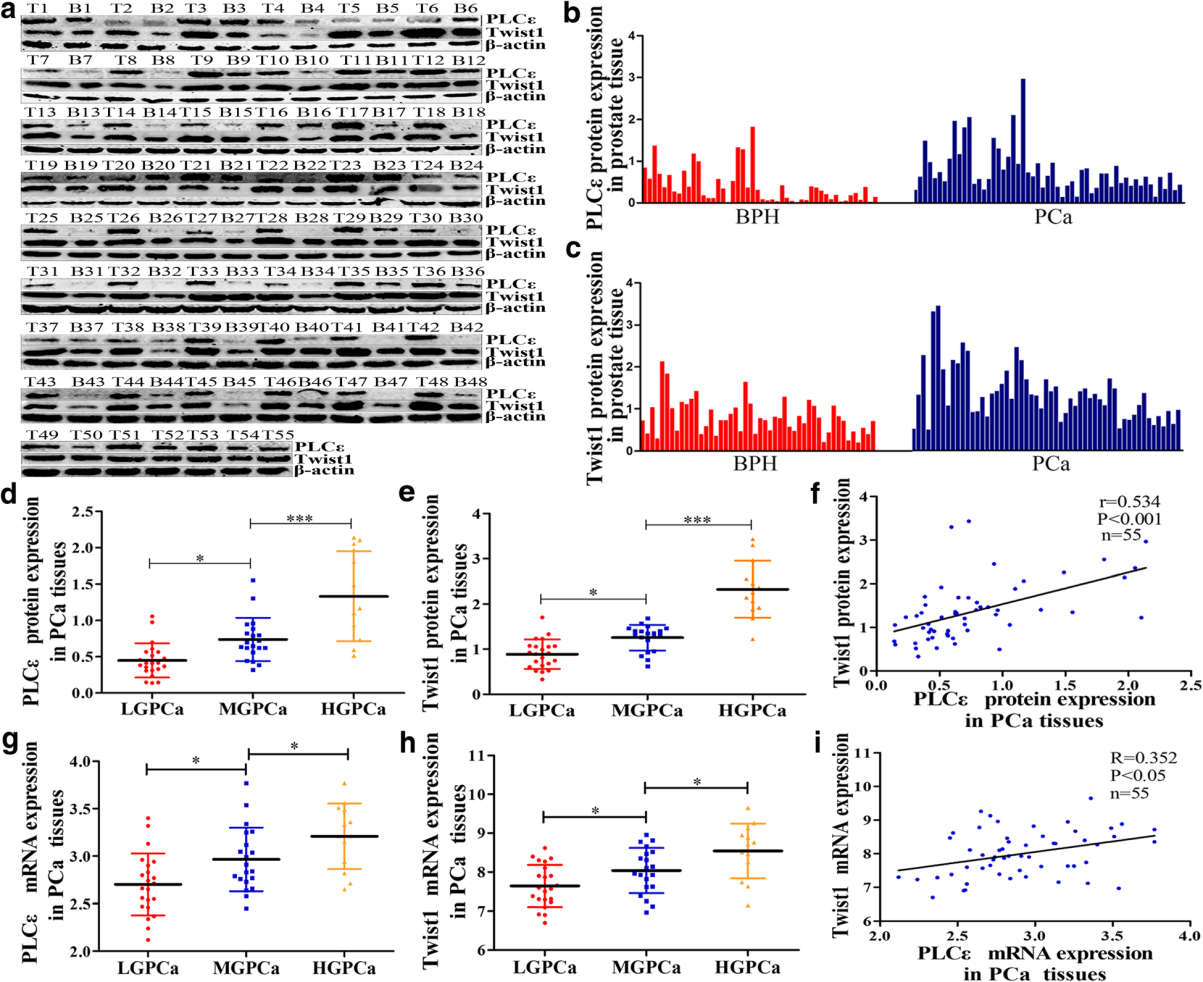
A association between PLCε and Twist1 expression in PCa patients. **a** PLCε and Twist1 protein expression in 55 PCa tissue samples (T) and 48 BPH samples (B) were assayed by western blotting analysis. **b**, **c** PLCε and Twist1 protein expression in tissues were quantified as mean optical density. **d**, **e** Average PLCε and Twist1 protein expression of different grades PCa. **f** Correlation curve of PCa PLCε protein versus corresponding Twist1. **g**, **h** Average PLCε and Twist1 mRNA expression of different grades PCa. **i** Correlation curve of PCa PLCε mRNA versus corresponding Twist1. Data were represented as the means ± SD. *P < 0.05, **P < 0.01

Fifty-five PCa tissue samples from patients treated with radical prostatectomy, and 48 BPH samples as controls, were tested for the presence of PLCε and Twist1. IHC analysis showed that approximately 70.9% (39/55) of the PCa samples were positive for PLCε staining, vs. 29.1% (16/55) of the BPH samples. Twist1 staining was positive in 85.5% (47/55) of the PCa samples vs. 14.5% (8/55) of the BPH samples (Fig. 1g). Semi-quantitative staining scores revealed significantly increased PLCε and Twist1 in PCa tissues, compared with that in BPH tissues (Fig. 1h, i, *P* < 0.001). This finding was confirmed by western blot analyses (Fig. 2a-c).

A positive association between PLCε and Twist1 IHC staining scores by Pearson’s correlation analysis was observed (Fig. 1j, *n* = 103, *r* = 0.8037, *P* < 0.001). Semi-quantitative analysis showed that PLCε and Twist1 protein expression in PCa tissue increased with increases in the degree of malignancy (Fig. 2d, e). Furthermore, a positive statistical association was found between the protein levels of PLCε and Twist1 (Fig. 2f, *P* < 0.001). Similar findings were observed with PLCε and Twist1 mRNA expression in PCa tissue (Fig. 2g-i). These results also demonstrated that the expression of PLCε and Twist1 was significantly associated with the Gleason grade in various clinicopathological characteristics of PCa patients (Table 1).

**Table 1:** PLCε and Twist1 in PCa tissues and clinicopathological parameters

	NO. specimens(%)	PLCε staining			Twist1 staining		
		Positive	Negative	*P* value	Positive	Negative	*P* value
Total	55 (100)	39 (70.9)	16 (29.1)		47 (85.5)	8 (14.5)	
Age (year)							
<60	19 (34.5)	14 (25.4)	5 (9.1)	0.499	15 (27.2)	4 (7.3)	0.285
≥60	36 (65.5)	25 (45.5)	11 (20.0)		32 (58.2)	4 (7.3)	
Histological stage							
Ta-T2	17 (30.9)	8 (14.5)	9 (16.4)	0.012	10 (18.2)	7 (12.7)	0.001
T3-T4	38 (69.1)	31 (56.4)	7 (12.7)		37 (67.3)	1 (1.8)	
Gleason scroe							
<7	22 (40)	11 (25)	11 (25)	0.07	16 (29.1)	6 (10.9)	0.037
≥7	33 (60)	28 (42.5)	5 (7.5)		31 (56.4)	2 (3.6)	

**Fig. 3:**
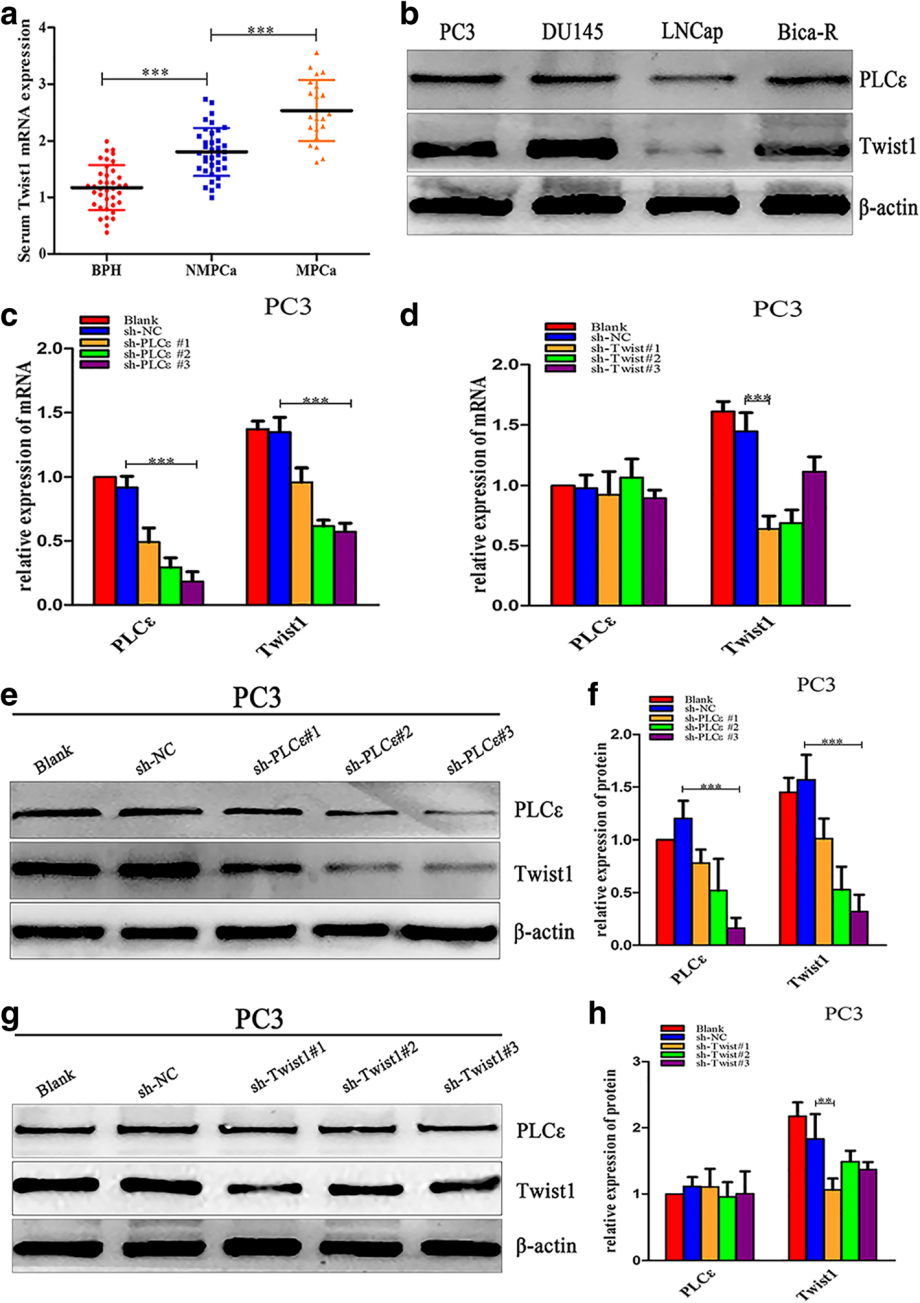
Knockdown of PLCε suppresses Twist1 mRNA and protein expression in PCa cell lines. **a** Serum Twist1 mRNA expression in BPH, metastatic (M) and non-metastatic (NM) PCa. **b** Western blotting analysis detected the expression of PLCε and Twist1 in four human PCa cell lines. **c**, **d** The mRNA expression of both PLCε and Twist1 after knockdown of PLCε or Twist1 in PC3 cells line. **e**, **f** The PC3 cells were infected with three lentiviral sh-PLCε, western blotting and protein quantification analyses were detected the expression of PLCε and Twist1. **g**, **h** The PC3 cells were transfected with three sh-Twist1plasmids, western blotting analysis of PLCε and Twist1. Proteins were quantified using image software. Data were represented as mean ± SD. of three individual experiments. *p < 0.05, **p < 0.01, and *** *p* < 0.001 vs. Controls

We next investigated whether high expression of Twist1 could be a prognostic marker of PCa due to its association with the metastasis of various cancers. Using a distinct group of clinical patient serum samples, we observed that serum Twist1 mRNA expression was higher in PCa than in BPH, and was also higher in metastatic than in non-metastatic PCa (Fig. 3a, *P* < 0.001). Furthermore, a high Twist1 mRNA serum level significantly correlated with the Gleason grade in various clinicopathological characteristics (Table 2).

**Table 2:** Twist1 in PCa patients serum and clinicopathological parameters

	No. specimens(%)	Twist1 mRNA		
		≥1.5	<1.5	*P* value
Histology				
BPH	39	9	30	0.000
PCa	58	50	8	
Age (years)				
<60	42	27	15	0.345
≥60	55	32	23	
Histological stage				
Ta-T2	59	27	32	0.002
T3-T4	38	32	6	
Gleason scroe				
<7	71	37	34	0.003
≥7	26	22	4	

### Knockdown of PLCε suppresses Twist1 mRNA and protein expression in PCa cell lines

Four human PCa cell lines (PC3, DU145, LNCap, and Bica-R) were examined by western blotting. The results showed that these celllines exhibited varying levels of PLCε and Twist1 (Fig. 3b), and in response to bicalutamide-resistance, Twist1 expression increased in LNCap. PLCε expression was also upregulated in Bica-R cells, further indicating a possible correlation between PLCε and Twist1 levels. Therefore, subsequent experiments generally used the three cells lines PC3, DU145, and Bica-R.

To silence PLCε, PCa cells were infected with three lentiviral short hairpin (sh) RNAs specific for PLCε (LV-sh-PLCε#1, LV-sh-PLCε#2, and LV-sh-PLCε#3). LV-sh-PLCε#3 markedly reduced the mRNA and protein expression of both PLCε and Twist1 (Fig. 3c, e, f).We also found that vector-sh-Twist1#1 effectively downregulated Twist1 mRNA and protein expression without affecting PLCε expression (Fig. 3d, g, h).

### PLCε expression negatively correlates with PGC-1α-mediated mitochondrial oxidative metabolism and uncoupling through Twist1

**Fig. 4:**
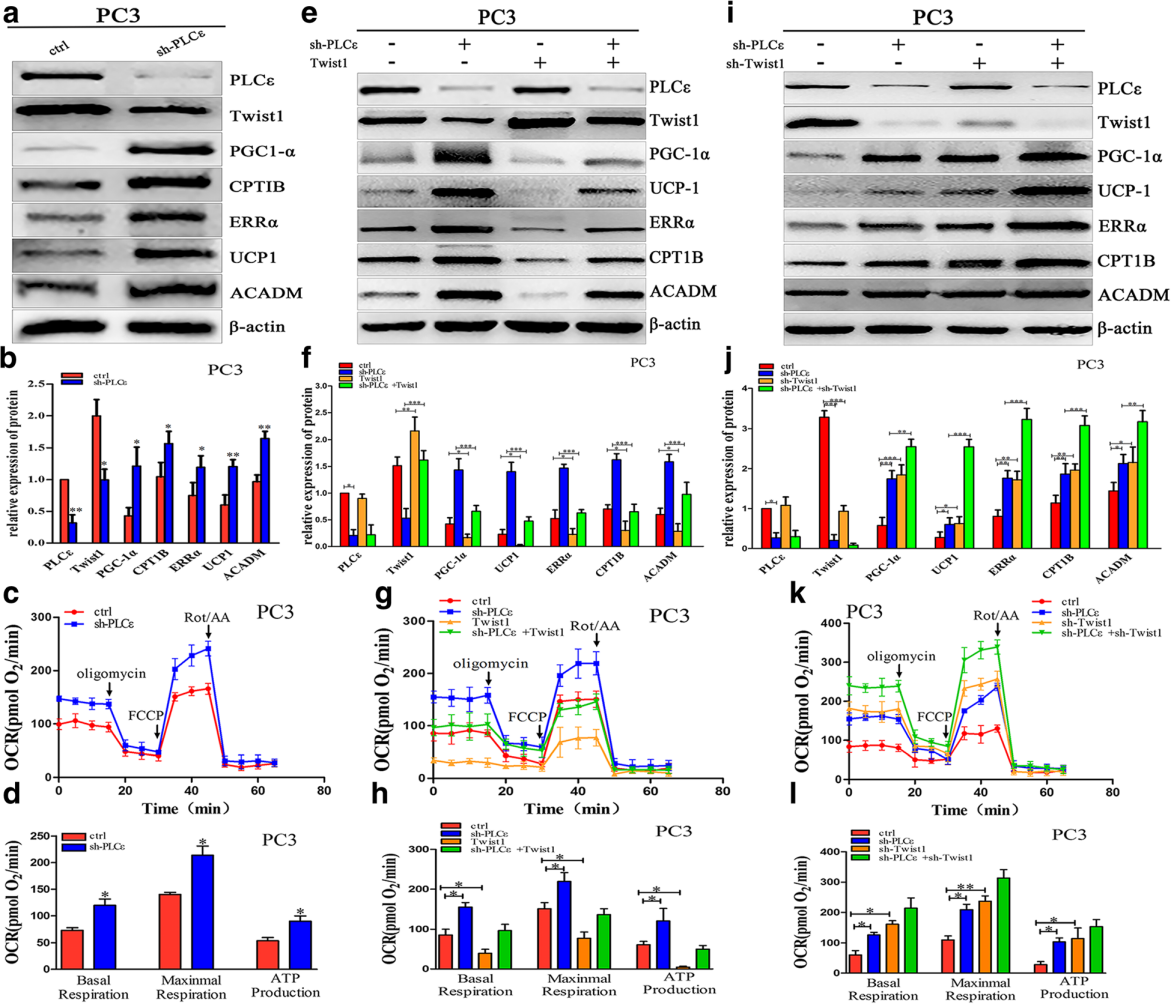
PLCε expression negatively correlates with PGC-1α-mediated mitochondrial oxidative metabolism and uncoupling through Twist1 in PC3 cells. **a**, **e** and **i** Western blotting analysis detected the expression of PLCε, Twist1, PGC-1α, CPT1B, ERRα, UCP-1, and ACADM after infected with lentiviral sh-PLCε or transfected with DDK-Twist1 or sh-Twist1 plasmids in PC3 cells. **b**, **f** and **j** Proteins were quantified using image software normalized against β-actin. **c**, **g** and **k** Seahorse tracing of the oxygen consumption rate in PC3 cells, followed by mitochondrial stress test as described in methods. **d**, **h** and **l** Bar graphs of means ± SD of the basal and maximal respiration and ATP production in PC3 cells. Data were represented as mean ± SD. of three individual experiments. *p < 0.05, **p < 0.01, and *** *p* < 0.001 vs. Controls

We next investigated whether downregulating PLCε in PCa cells had any effects on mitochondrial oxidative metabolism and uncoupling through inhibition of Twist1 expression. As shown in Fig. 4a and b, and Additional file 1: Figure S2a and b, stable knockdown of PLCε increased the expression of PGC-1α, CPT1B, ERRα, UCP-1, and ACADM, and decreased the expression of Twist1.

Based on these findings, we examined the mitochondrial activity of PCa cell lines by measuring the OCR. The results showed that knockdown of PLCε increased the OCR and ATP production in PCa cells (Fig. 4c, d and Additional file 1: Figure S2c, d). We hypothesized that PLCε regulated mitochondrial oxidative metabolism and uncoupling specifically through Twist1. Western blotting and OCR analyses showed that overexpressing Twist1 in sh-PLCε-transfected PCa cells abolished the sh-PLCε-mediated increases in metabolism, uncoupling, OCR, and ATP production (Fig. 4e-h and Additional file 1: Figure S2e-h). Interestingly, the combination of sh-PLCε and sh-Twist1 promoted metabolism and uncoupling significantly more than either shRNA alone (Fig. 4i-l and Additional file 1: Figure S2i-l). Together, these data support our hypothesis that the PLCε/Twist1 axis suppresses PCa cell mitochondrial oxidative metabolism and uncoupling.

### PLCε depletion decreases phosphorylation of Twist1 on serine 68 and reduces stability of Twist1 protein via MAPKs

At the gene level, the expression of Twist1 is modulated by several upstream regulators through multiple pathways, depending on the cancer type and tissue context [35]. For example, signal transducer and activator of transcription 3 binds directly to the human Twist1 promoter and activates its transcriptional activity in human breast cancer, hepatocellular carcinoma, and gastric cancer cell lines [36–38]. Moreover, NF-κB, Ras, and transforming growth factor-β signaling pathways enhance Twist1 expression [39–41]. At the post-translational level, the MAPK pathway decreases E3-mediated ubiquitination and stabilizes Twist1 without altering mRNA expression through a considerable increase in Twist1 serine68 phosphorylation in breast cancer cells [42]. PLCε can also regulate Ras expression [16].

**Fig. 5:**
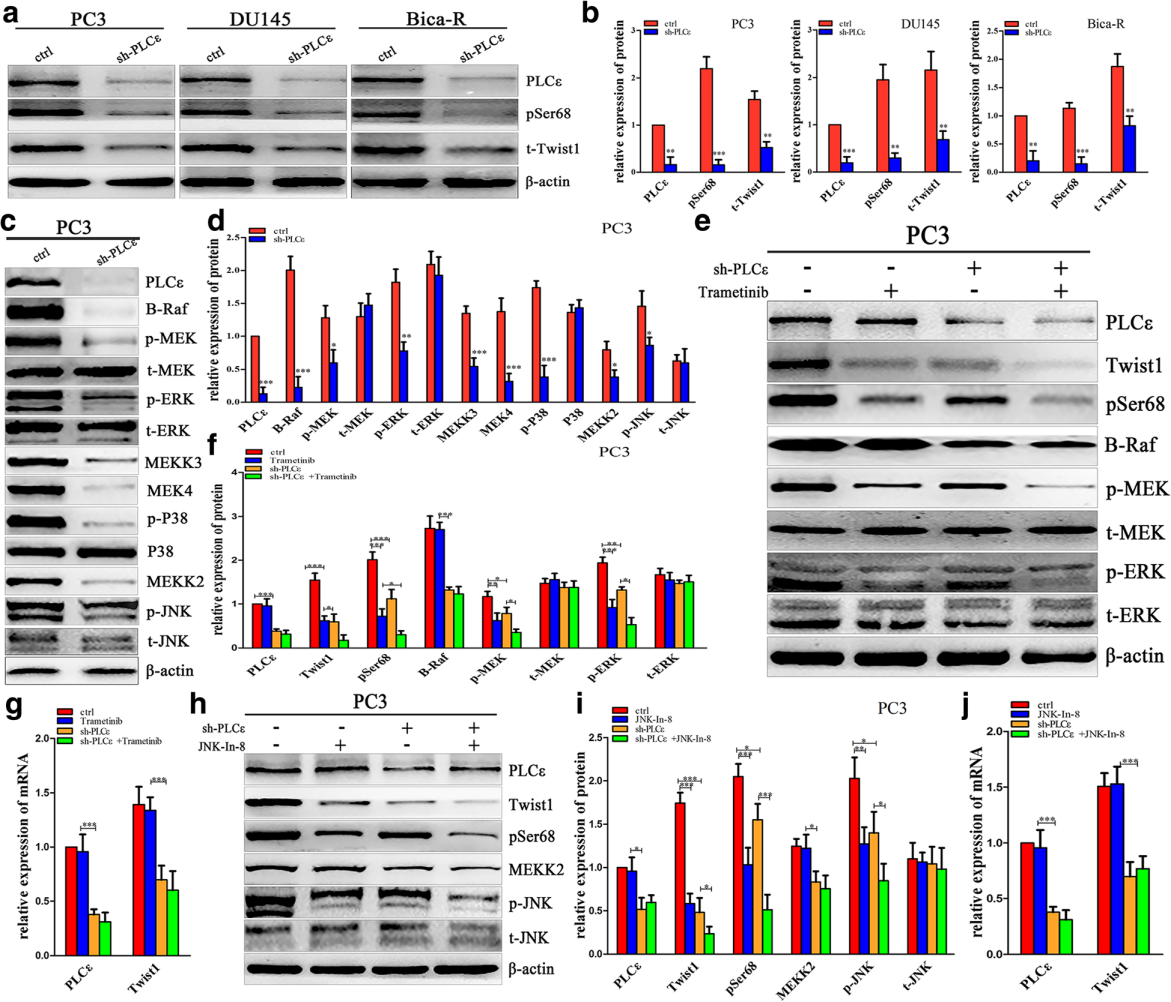
PLCε depletion decreases the phosphorylation of Twist1 on serine 68 and reduces stability of Twist1 protein via MAPKs. **a**, **b** Western blotting and protein quantification analyses detected the expression of PLCε, phosphorylation of serine 68 of Twist1 (pSer68) and Twist1 after knockdown PLCε in PCa cells. **c**, **d** Western blotting and protein quantification analyses detected the expression of B-Raf, p-MEK, t-MEK, p-ERK, t-ERK, MEKK3, MEK4, p-P38, P38, MEKK2, p-JNK and t-JNK in sh-PLCε-infected PC3 cells. **e**, **f** Western blotting and protein quantification analyses detected the expression of PLCε, Twist1, pSer68, B-Raf, p-MEK, t-MEK, p-ERK and t-ERK in PC3 cells treated with 1 nM trametinib. **g**, **j** Relative mRNA of PLCε and Twist1 in PC3 cells treated with trametinib or JNK-In-8. **h**, **i** Western blotting and protein quantification analyses detected the expression of PLCε, Twist1, pSer68, MEKK2, p-JNK and t-JNK in PC3 cells treated with 5 nM JNK-In-8. Data were represented as mean ± SD. of three individual experiments. *p < 0.05, **p < 0.01, and *** *p* < 0.001 vs. Controls

To explore the mechanisms through which PLCε modulate Twist1 expression, we analyzed the MAPK signaling pathway, the main pathway upstream of Twist. Western blotting showed decreased levels of Twist 1 and phosphorylated (p)Twist1 (Fig. 5a, b), as well as decreased p-MEK, p-ERK, p-P38, and p-c-Jun N-terminal kinase (JNK), and some key molecules in the MAPK pathway (Fig. 5c, d and Additional file 1: Figure S3a, b) in PCa cells treated with PLCε-shRNA. We subsequently utilized 1 nM trametinib (a specific inhibitor of MEK), 5 nM JNK-In-8 (a specific inhibitor of JNK), or 0.5 μM SB203580 (a specific inhibitor of P38 MAPK) to evaluate whether these signaling pathways were involved in PLCε-mediated Twist1 expression. A concentration achieving 50% inhibition efficiency was selected for each inhibitors (Additional file 1: Figure S3c-e). The results showed that all three inhibitors effectively antagonized their corresponding targets and decreased Twist1 protein levels in the absence or presence of PLCε but had little effect on Twist1 mRNA levels (Fig. 5e-j, Additional file 1: Figures S3f-j and S4a-c).

**Fig. 6:**
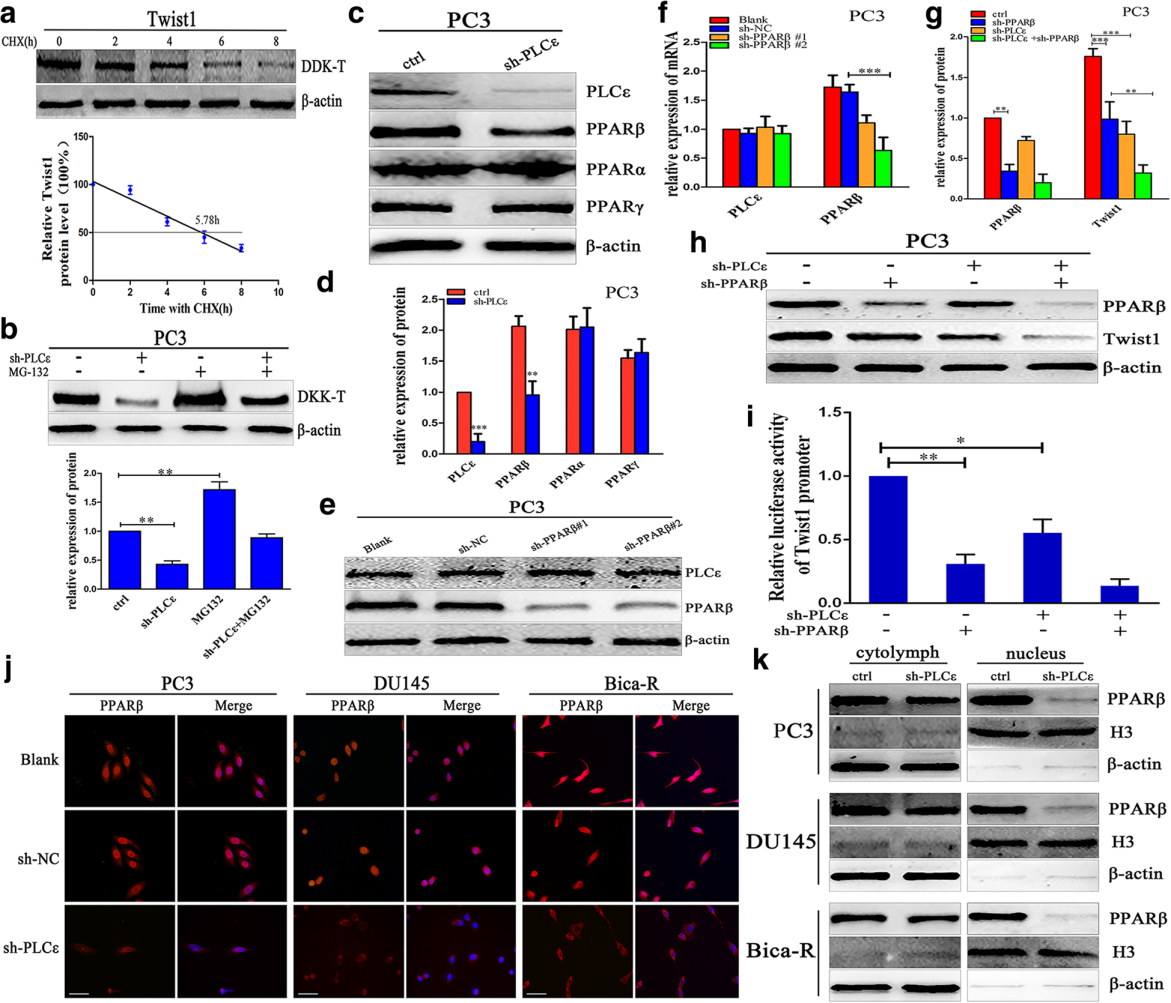
PLCε regulates Twist1 through nuclear translocation of PPARβ. **a** PC3 cells were transfected with DDK-Twist1 plasmid. After 12 h, cells were treated with 100 μM cycloheximide (CHX) for time periods as indicated. Western blotting analysis was conducted with DDK and β-actin antibodies. Densitometric values were determined and presented. The half-life (50%) of DDK-Twist1 was indicated. **b** PC3 cells were infected with sh-PLCε lentiviral and treated with 25 μM MG-132 for 24 h, and cell lysates were collected for western blotting and protein quantification analyses. **c**, **d** Western blotting and protein quantification analyses detected the expression of PLCε, PPARβ, PPARα and PPARγ in sh-PLCε-infected PC3 cells. **e**, **f** The protein and mRNA expression of PLCε and PPARβ after knockdown of PPARβ in PC3 cells line. **g**, **h** Western blotting and protein quantification analyses detected the expression of PPARβ and Twist1 in sh-PLCε-infected PC3 cells. **i** Twist1 promoter transcriptional activity determined by luciferase assay in PC3 cells. **j** Immunofluorescence staining demonstrated PPARβ intracellular distribution in three PCa cell lines.Magnification × 400. Bars = 50 μm. **k** A nuclear-cytoplasmic separation was performed in PCa cells. Western blotting analysis of each fraction detected the expression of PPARβ. β-actin and Histone (H) 3 were used as an internal control. Data were represented as mean ± SD. of three individual experiments. *p < 0.05, **p < 0.01, and *** *p* < 0.001 vs. Controls

Finally, we assessed the stability of the Twist1 protein in PC3 cells after blocking protein synthesis with 100 μM cycloheximide. The half-life of Twist1 in DDK-Twist1-transfected PC3 cells was 5.78 h (Fig. 6a). This suggests that Twist1 protein has a short half-life and its stability is affected by ubiquitin modification. Interestingly, sh-PLCε-mediated inhibition of Twist1 protein levels could be reversed with 25 μM MG132 (a proteasome inhibitor) (Fig. 6b). Collectively, these finding indicated that the regulation of Twist1 by PLCε is probably proteasome-dependent, and that PLCε regulates Twist1 proteasomal degradation via MAPKs.

### PLCε regulates Twist1 through nuclear translocation of PPARβ

In PCa cells, knockdown of PLCε decreased the PPARβ protein level but had little effect on PPARα and PPARγ (Fig. 6c, d and Additional file 1: Figure S4d, e). PCa cells were transfected with two shRNAs specific for PPARβ (vector-sh-PPARβ#1 and vector-sh-PPARβ#2). Vector-sh-PPARβ#2 effectively downregulated PPARβ mRNA and protein expression (Fig. 6e, f and Additional file 1: Figure S4f). Interestingly, the combination of sh-PLCε and sh-PPARβ inhabited Twist1 mRNA and protein expression significantly more than either shRNA alone (Fig. 6g, h and Additional file 1: Figure S5c-e). PPARβ is a transcription factor that mediates lipid and fatty acid metabolism. Immunofluorescence staining showed that PLCε depletion markedly decreased the PPARβ signal in the nucleus (Fig. 6j). To confirm the distribution of PPARβ within the nucleus, we performed a nuclear-cytoplasmic separation. Western blotting of each fraction showed that the nuclear distribution of PPARβ was notably decreased in PCa cells (Fig. 6k). We surmised that PLCε depletion inhibits PPARβ by crippling its nuclear translocation.

**Fig. 7:**
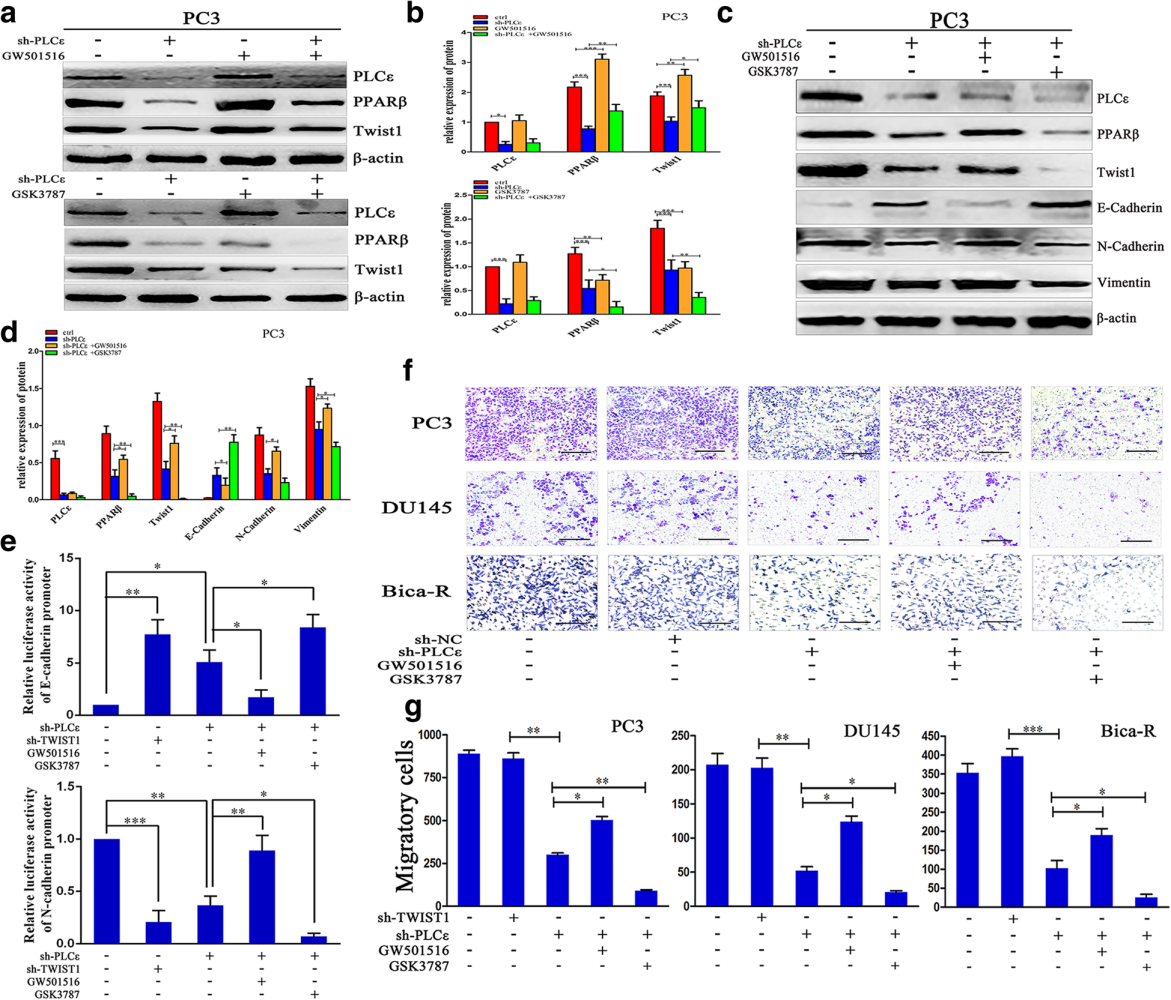
PLCε depletion inhibits EMT via PPARβ-mediated Twist1 downregulation. **a**, **b** Treatment of sh-PLCε-infected PC3 cells with 1 nM GW501516 and 1 μM GSK3787, cell lysates were collected for western blotting and protein quantification analyses. **c**, **d** Protein lysates from sh-PLCε-infected PC3 cells treated with GW501516 and GSK3787 were collected for western blotting and protein quantification analyses. **e** E-cadherin and N-cadherin transcriptional activity determined by luciferase assay in PC3 cells. **f**, **g** Transwell assays were employed to test the cell migratory capacity of PCa cell lines. Magnification × 200. Bars = 500 μm. Data were represented as mean ± SD. of three individual experiments. *p < 0.05, **p < 0.01, and *** *p* < 0.001 vs. Controls

The Twist1 luciferase reporter assay showed that PLCε and PPARβ knockdown suppressed Twist1 activities in PC3 cells. This indicated that PPARβ is a direct transcriptional target of Twist1 (Fig. 6i). Treatment of sh-PLCε-transfected PCa cells with1 nM GW501516, a highly selective agonist of PPARβ, prevented the PLCε depletion-mediated decreases in Twist1 mRNA and protein, while GSK3787 (1 μM), an inhibitor of PPARβ, enhanced PLCε depletion-mediated Twist1 repression (Fig. 7a, b and Additional file 1: Figure S5a-c).

To further support the hypothesis that PLCε promotes Twist1 transcription though PPARβ, assays were performed in PC3 cells with E-cadherin or N-cadherin promoter-driven luciferase reporters after depletion of PLCε or Twist1. The results demonstrated that PLCε and Twist1 knockdown enhanced E-cadherin and decreased N-cadherin reporter activities. Furthermore, GW501516 prevented and GSK3787 enhanced these effects (Fig. 7e). Collectively, these results indicate that PLCε induces Twist1 mRNA, protein, and transcriptional activity through PPARβ.

### PLCε depletion inhibits EMT via PPARβ-mediated Twist1 downregulation

To investigate whether PLCε regulates Twist1-mediated EMT and cell migration, western blotting analyses were performed. The results revealed that PLCε depletion inhibited the expression of Twist1, PPARβ, N-cadherin, and vimentin, and increased the expression of E-cadherin. GW501516 antagonized and GSK3787 promoted the effects of PLCε depletion in PC3 and DU145 cells (Fig. 7c, d and Additional file 1: Figure S5f, g). Finally, Transwell migration assays revealed that PLCε depletion inhibited the migration of PCa cells, and GW501516 antagonized and GSK3787 promoted this effect (Fig. 7f, g).

### PLCε depletion enhances the inhibitory effect of Twist1 knockdown on PCa tumorigenesis in vivo

**Fig. 8:**
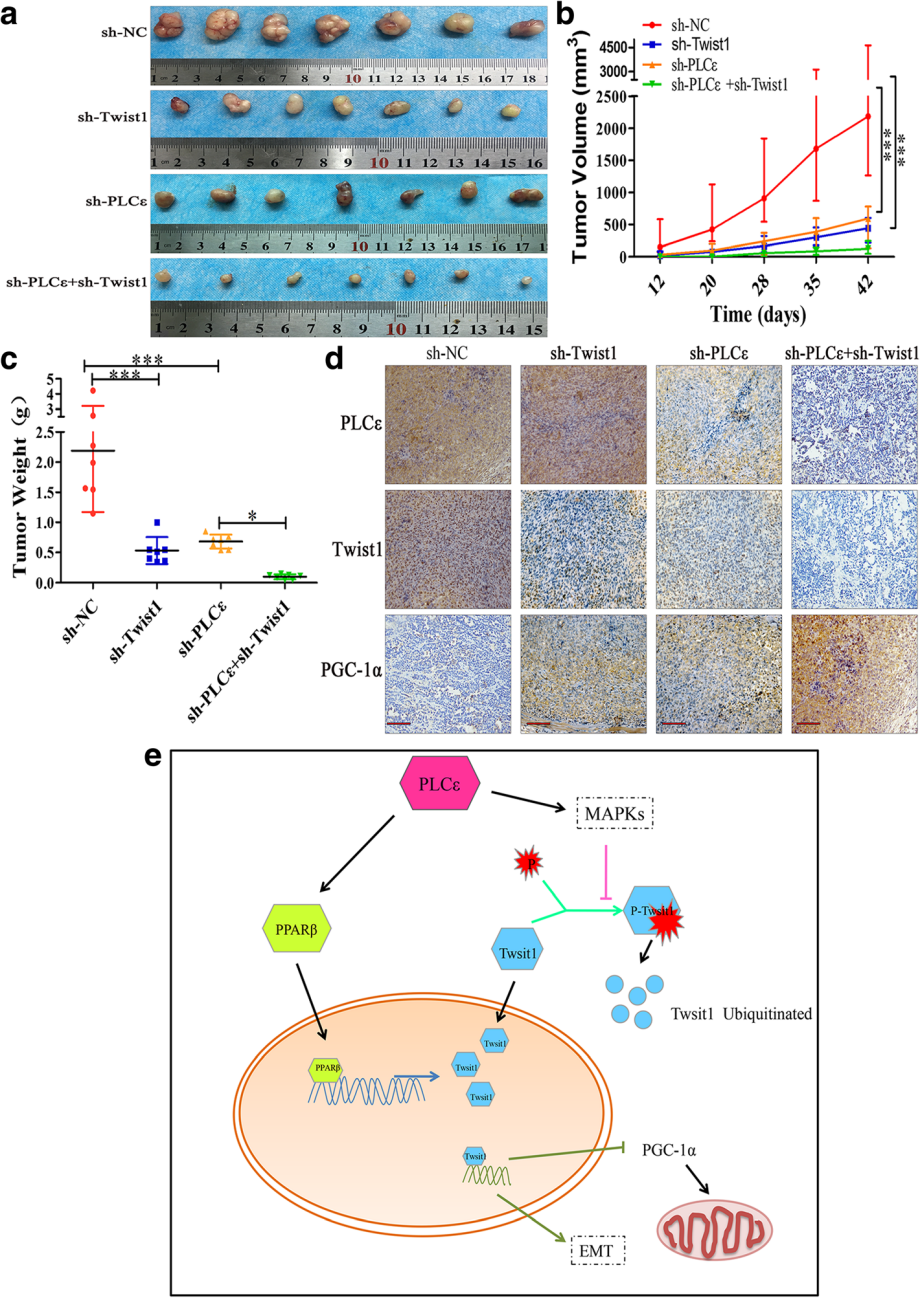
PLCε depletion enhanced the inhibitory effect of Twist1 knockdown on PCa tumorigenesis in vivo. **a** The photographs of dissected tumors from nude mice were shown. **b**, **c** The growth curves and the average weights of tumors from nude mice after injection with PC3 cells infected sh-NC, sh-PLCε and sh-Twist1. Data were represented as mean ± SD. *p < 0.05. **d** IHC staining of PLCε, Twist1 and PGC-1α in tumor tissues from mice. Magnification × 200. Bars = 100 μm. **e** A mechanism for PLCε depletion-mediated Twist1 downregulation. Schematic diagram describing the functional significance of PLCε in PCa cells. PLCε may regulate Twist1 expression through MAPK-mediated ubiquitination and PPARβ-mediated transcription

An in vivo nude mouse tumor xenograft assay was conducted to verify the role of PLCε in tumor formation. Consistent with the in vitro findings, the PLCε- and Twist1-depleted groups both displayed less prominent tumorigenesis than the sh-NC group; the mean tumor volumes and weights were both lower in the PLCε- and Twist1-depleted groups than in the sh-NC group. Combined knockdown inhibited the growth and weight of tumors more than either knockdown alone (Fig. 8a-c). In addition, IHC analyses of the mouse tumors revealed that PLCε and Twist1 were expressed at low levels and PGC-1α was expressed at a high level in the sh-PLCε group compared to that in the sh-NC group. Downregulation of Twist1 alone decreased Twist1 and increased PGC-1α levels, while the combined treatment group expressed less PLCε and Twist1, and more PGC-1α than other three groups (Fig. 8d). These results support the tumor suppressor function of PLCε depletion in PCa.

## Discussion

The results presented here reveal that PLCε exerts tumor promotional activity in PCa cell lines through effects on mitochondrial oxidative metabolism. We demonstrated that PLCε depletion could regulate Twist1 mRNA levels and protein stability through two possible mechanisms, and thus promote PGC-1α-mediated mitochondrial oxidative metabolism. Moreover, the oxidative metabolic program elicited by PGC-1α can prevent tumor growth and migration.

PLCε and Twist1are associated with multiple types of cancers, including PCa [43–45]. We used patient prostate and serum samples to provide a comprehensive analysis of PLCε and Twist1 expression in normal (i.e., BPH) and cancerous (i.e., PCa) tissues. The results indicated that high expression of PLCε in PCa tissues correlated with low survival and a high Gleason score. Significantly elevated Twist1 expression in prostate tissues and serum from PCa patients also correlated significantly with low survival and a high Gleason score. Moreover, a statistical association was found between PLCε and Twist1 expression in PCa. Thus, measurements of PLCε and Twist1 might have diagnostic and/or prognostic value in PCa.

PLCε is required for the calcium release mechanism [46] that is responsible for the activity of the mTOR and MAPK pathways. Importantly, it is widely believed that mitochondrial oxidative metabolism is controlled by both the mTOR and MAPK pathways [47, 48]. Thus, we hypothesized that PLCε regulated mitochondrial oxidative metabolism in PCa. In our studies, we knocked down PLCε in PCa cell lines using lentiviral sh-PLCε. PLCε depletion decreased Twist1 mRNA and protein levels. This implied that PLCε may regulate Twist1 mRNA levels. PLCε depletion also increased the expression of PGC-1α, CPT1B, ERRα, UCP-1, and ACADM, and promoted the OCR. By transfecting cells with a Twist1 (Myc-DDK-tagged) vector and vector-sh-Twist1, we clearly found that PLCε regulated PCa mitochondrial oxidative metabolism by targeting Twist1.

MAPKs can promote breast tumor cell EMT and metastasis via phosphorylation and stabilization of Twist1, and PLCε can regulate key molecules involved in MAPK expression [42]. We observed that knocking down PLCε decreased p-Twist1expression significantly, and an inhibitor of the MAPK pathway suppressed both Twist1 and p-Twist1 levels. Interestingly, sh-PLCε-mediated inhibition of Twist1 protein levels could be reversed with MG132. These results suggest that PLCε depletion suppresses the phosphorylation of Twist1 and accelerates its ubiquitination, thus decreasing Twist1 protein levels via all three MAPK subfamily members.

Interestingly, PLCε depletion decreased Twist1 mRNA and protein levels, which implies that PLCε regulates Twist1 mRNA levels in another way. PPARβ, a member of the PPAR nuclear hormone receptor super family, is involved in brain lipid metabolism, proliferation of anterior fat cells, fat formation, embryonic inhibition, macrophage cholesterol homeostasis, and tumor formation after being activated by ligands [49]. Some studies suggest that PPARβ is highly expressed in PCa cells and is associated with cell cycle regulation and proliferation [50]. PLC influences the expression of PPARβ through Ca^2+^/cytosolic phospholipase A2, thus affecting glucose metabolism [51, 52]. Following PLCε knockdown, we observed a decreased level of PPARβ alone. Using GW501516 and GSK3787, an agonist and inhibitor of PPARβ, respectively, we showed that PLCε might regulate the transcription of Twist1, as well as Twist1-mediated EMT, cell migration, and transcriptional regulation; these are well-known features of Twist1 [35]. Importantly, immunofluorescence and western blot analyses indicated that PLCε depletion probably blocked PPARβ nuclear translocation.

## Conclusions

The results of our study have elucidated a probable role for PLCε in antagonizing mitochondrial oxidative metabolism in PCa. We also identified that these changes are mediated by Twist1. Moreover, PLCε may regulate Twist1 expression through several pathways, including MAPK-mediated ubiquitination and PPARβ-mediated transcription.

## Additional file

Additional file 1: **Figure S1.** High expression levels of PLCε and Twist1 in PCa tissues. (a) RNA-seq mRNA expression data from the TCGA and GTEx database was used to compare Twist1 expression between in PCa tumors (T) (*n* = 492) and their non-tumor counterparts (N) (*n* = 152). *p < 0.05. (b) Correlation between the expression of Twist1 and PLCε in PCa patients from cBioPortal database. **Figure S2.** PLCε expression negatively correlates with PGC-1α-mediated mitochondrial oxidative metabolism and uncoupling through Twist1 in DU145 and Bica-R cells. **Figure S3.** PLCε depletion decreases phosphorylation of Twist1 on serine 68 and reduces stability of Twist1 protein via MAPKs in DU145 and Bica-R cells. **Figure S4** PLCε depletion decreases Twist1 protein via MAPKs and alleviates the PPARβ expression. **Figure S5** PLCε depletion decreases Twist1 by PPARβ. (DOCX 3776 kb)

## Data Availability

Literature collection was performed using PubMed. Statistical analysis were executed by using SPSS 17.0 software (IBM, Chicago, IL, USA) and GraphPad (Prism 5.0). Raw and processed data are stored in corresponding author and are available upon request. The RNAseq datasets and clinical information of Twist1 and PLCε in PCa were retrieved from TCGA database (http://www.cancergenome.nih.gov), cBioPortal for Cancer Genomics (http://www.cbioportal.org/), GEO Datasets (https://www.ncbi.nlm.nih.gov/gds) and GTEx portal (https://www.gtexportal.org).

## Abbreviations

Bica-R: Bicalutamide-resistant
BPH: Benign prostatic hypertrophy
CHX: Cycloheximide
EMT: Epithelial-to-mesenchymal transition
ETC: Electron transport chain
FCCP: Luoro-carbonyl cyanide phenylhydrazone
IHC: Immunohistochemical
JNK: Jun N-terminal kinase
MAPKs: Mitogen-activated protein kinases
OCR: Oxygen consumption rates
PCa: Prostate cancer
PGC-1α: Peroxisome proliferator-activated receptor gamma co-activator 1 alpha
PLC: Phospholipase C
PPAR: Peroxisome proliferator activated receptor
Rot/AA: Antimycin A/rotenone
sh: Short hairpin

## References

[1] Beltran H, Tomlins S, Aparicio A, Arora V, Rickman D, Ayala G, Huang J, True L, Gleave ME, Soule H (2014). Aggressive variants of castration-resistant prostate cancer. Clin Cancer Res.

[2] Bostwick DG (1997). Staging prostate cancer--1997: current methods and limitations. Eur Urol.

[3] Herst P. M., Grasso C., Berridge Michael V. (2018). Metabolic reprogramming of mitochondrial respiration in metastatic cancer. Cancer and Metastasis Reviews.

[4] Wallace M, Metallo CM (2016). PGC1alpha drives a metabolic block on prostate cancer progression. Nat Cell Biol.

[5] Liberti MV, Locasale JW (2016). The Warburg effect: how does it benefit Cancer cells?. Trends Biochem Sci.

[6] Vander Heiden MG, Cantley LC, Thompson CB (2009). Understanding the Warburg effect: the metabolic requirements of cell proliferation. Science..

[7] Puigserver P, Wu Z, Park CW, Graves R, Wright M, Spiegelman BM (1998). A cold-inducible coactivator of nuclear receptors linked to adaptive thermogenesis. Cell..

[8] Michael LF, Wu Z, Cheatham RB, Puigserver P, Adelmant G, Lehman JJ, Kelly DP, Spiegelman BM (2001). Restoration of insulin-sensitive glucose transporter (GLUT4) gene expression in muscle cells by the transcriptional coactivator PGC-1. Proc Natl Acad Sci U S A.

[9] Lin J, Handschin C, Spiegelman BM (2005). Metabolic control through the PGC-1 family of transcription coactivators. Cell Metab.

[10] Audet-Walsh E, Papadopoli DJ, Gravel SP, Yee T, Bridon G, Caron M, Bourque G, Giguere V, St-Pierre J (2016). The PGC-1alpha/ERRalpha Axis represses one-carbon metabolism and promotes sensitivity to anti-folate therapy in breast Cancer. Cell Rep.

[11] Torrano V, Valcarcel-Jimenez L, Cortazar AR, Liu X, Urosevic J, Castillo-Martin M, Fernandez-Ruiz S, Morciano G, Caro-Maldonado A, Guiu M (2016). The metabolic co-regulator PGC1alpha suppresses prostate cancer metastasis. Nat Cell Biol.

[12] Schopf B, Schafer G, Weber A, Talasz H, Eder IE, Klocker H, Gnaiger E (2016). Oxidative phosphorylation and mitochondrial function differ between human prostate tissue and cultured cells. FEBS J.

[13] Bunney TD, Katan M (2006). Phospholipase C epsilon: linking second messengers and small GTPases. Trends Cell Biol.

[14] Bunney TD, Katan M (2011). PLC regulation: emerging pictures for molecular mechanisms. Trends Biochem Sci.

[15] Singer WD, Brown HA, Sternweis PC (1997). Regulation of eukaryotic phosphatidylinositol-specific phospholipase C and phospholipase D. Annu Rev Biochem.

[16] Jin TG, Satoh T, Liao Y, Song C, Gao X, Kariya K, Hu CD, Kataoka T (2001). Role of the CDC25 homology domain of phospholipase Cepsilon in amplification of Rap1-dependent signaling. J Biol Chem.

[17] Sorli SC, Bunney TD, Sugden PH, Paterson HF, Katan M (2005). Signaling properties and expression in normal and tumor tissues of two phospholipase C epsilon splice variants. Oncogene..

[18] Wang X, Zhou C, Qiu G, Yang Y, Yan D, Xing T, Fan J, Tang H, Peng Z (2012). Phospholipase C epsilon plays a suppressive role in incidence of colorectal cancer. Med Oncol.

[19] Danielsen SA, Cekaite L, Agesen TH, Sveen A, Nesbakken A, Thiis-Evensen E, Skotheim RI, Lind GE, Lothe RA (2011). Phospholipase C isozymes are deregulated in colorectal cancer--insights gained from gene set enrichment analysis of the transcriptome. PLoS One.

[20] Bunney TD, Baxendale RW, Katan M (2009). Regulatory links between PLC enzymes and Ras superfamily GTPases: signalling via PLCepsilon. Adv Enzym Regul.

[21] Zhou RM, Li Y, Wang N, Liu BC, Chen ZF, Zuo LF (2012). PLC-epsilon1 gene polymorphisms significantly enhance the risk of esophageal squamous cell carcinoma in individuals with a family history of upper gastrointestinal cancers. Arch Med Res.

[22] Wang Y, Wu X, Ou L, Yang X, Wang X, Tang M, Chen E, Luo C (2015). PLCepsilon knockdown inhibits prostate cancer cell proliferation via suppression of notch signalling and nuclear translocation of the androgen receptor. Cancer Lett.

[23] Wang LD, Zhou FY, Li XM, Sun LD, Song X, Jin Y, Li JM, Kong GQ, Qi H, Cui J (2010). Genome-wide association study of esophageal squamous cell carcinoma in Chinese subjects identifies susceptibility loci at PLCE1 and C20orf54. Nat Genet.

[24] Thisse B, el Messal M, Perrin-Schmitt F (1987). The twist gene: isolation of a Drosophila zygotic gene necessary for the establishment of dorsoventral pattern. Nucleic Acids Res.

[25] Yang J, Mani SA, Donaher JL, Ramaswamy S, Itzykson RA, Come C, Savagner P, Gitelman I, Richardson A, Weinberg RA (2004). Twist, a master regulator of morphogenesis, plays an essential role in tumor metastasis. Cell..

[26] Martin A, Cano A (2010). Tumorigenesis: Twist1 links EMT to self-renewal. Nat Cell Biol.

[27] Mani SA, Guo W, Liao MJ, Eaton EN, Ayyanan A, Zhou AY, Brooks M, Reinhard F, Zhang CC, Shipitsin M (2008). The epithelial-mesenchymal transition generates cells with properties of stem cells. Cell..

[28] Qin Q, Xu Y, He T, Qin C, Xu J (2012). Normal and disease-related biological functions of Twist1 and underlying molecular mechanisms. Cell Res.

[29] Lee KE, Bar-Sagi D (2010). Oncogenic KRas suppresses inflammation-associated senescence of pancreatic ductal cells. Cancer Cell.

[30] Parajuli P, Kumar S, Loumaye A, Singh P, Eragamreddy S, Nguyen TL, Ozkan S, Razzaque MS, Prunier C, Thissen JP (2018). Twist1 Activation in Muscle Progenitor Cells Causes Muscle Loss Akin to Cancer Cachexia. Dev Cell.

[31] Pan D, Fujimoto M, Lopes A, Wang YX (2009). Twist-1 is a PPARdelta-inducible, negative-feedback regulator of PGC-1alpha in brown fat metabolism. Cell..

[32] Quan Z, He Y, Luo C, Xia Y, Zhao Y, Liu N, Wu X (2017). Interleukin 6 induces cell proliferation of clear cell renal cell carcinoma by suppressing hepaCAM via the STAT3-dependent up-regulation of DNMT1 or DNMT3b. Cell Signal.

[33] Du Z, Li L, Sun W, Wang X, Zhang Y, Chen Z, Yuan M, Quan Z, Liu N, Hao Y (2018). HepaCAM inhibits the malignant behavior of castration-resistant prostate cancer cells by downregulating notch signaling and PF-3084014 (a gamma-secretase inhibitor) partly reverses the resistance of refractory prostate cancer to docetaxel and enzalutamide in vitro. Int J Oncol.

[34] Alexander NR, Tran NL, Rekapally H, Summers CE, Glackin C, Heimark RL (2006). N-cadherin gene expression in prostate carcinoma is modulated by integrin-dependent nuclear translocation of Twist1. Cancer Res.

[35] Zhao Z, Rahman MA, Chen ZG, Shin DM (2017). Multiple biological functions of Twist1 in various cancers. Oncotarget..

[36] Cheng GZ, Zhang WZ, Sun M, Wang Q, Coppola D, Mansour M, Xu LM, Costanzo C, Cheng JQ, Wang LH (2008). Twist is transcriptionally induced by activation of STAT3 and mediates STAT3 oncogenic function. J Biol Chem.

[37] Zhang C, Guo F, Xu G, Ma J, Shao F (2015). STAT3 cooperates with twist to mediate epithelial-mesenchymal transition in human hepatocellular carcinoma cells. Oncol Rep.

[38] Hsu KW, Hsieh RH, Huang KH, Fen-Yau Li A, Chi CW, Wang TY, Tseng MJ, Wu KJ, Yeh TS (2012). Activation of the Notch1/STAT3/twist signaling axis promotes gastric cancer progression. Carcinogenesis..

[39] Jiang J, Kosman D, Ip YT, Levine M (1991). The dorsal morphogen gradient regulates the mesoderm determinant twist in early Drosophila embryos. Genes Dev.

[40] Satoh K, Hamada S, Kimura K, Kanno A, Hirota M, Umino J, Fujibuchi W, Masamune A, Tanaka N, Miura K (2008). Up-regulation of MSX2 enhances the malignant phenotype and is associated with twist 1 expression in human pancreatic cancer cells. Am J Pathol.

[41] Thuault S, Valcourt U, Petersen M, Manfioletti G, Heldin CH, Moustakas A (2006). Transforming growth factor-beta employs HMGA2 to elicit epithelial-mesenchymal transition. J Cell Biol.

[42] Hong J, Zhou J, Fu J, He T, Qin J, Wang L, Liao L, Xu J (2011). Phosphorylation of serine 68 of Twist1 by MAPKs stabilizes Twist1 protein and promotes breast cancer cell invasiveness. Cancer Res.

[43] Han KY, Chen PN, Hong MC, Hseu YC, Chen KM, Hsu LS, Chen WJ (2018). Naringenin attenuated prostate Cancer invasion via reversal of epithelial-to-mesenchymal transition and inhibited uPA activity. Anticancer Res.

[44] Yang Y, Wu XH (2017). Study on the influence of metformin on castration-resistant prostate cancer PC-3 cell line biological behavior by its inhibition on PLCepsilon gene-mediated Notch1/Hes and androgen receptor signaling pathway. Eur Rev Med Pharmacol Sci.

[45] Wang X, Fan Y, Du Z, Fan J, Hao Y, Wang J, Wu X, Luo C (2018). Knockdown of phospholipase Cepsilon (PLCepsilon) inhibits cell proliferation via phosphatase and Tensin homolog deleted on chromosome 10 (PTEN)/AKT signaling pathway in human prostate Cancer. Med Sci Monit.

[46] Kondratskyi A, Yassine M, Kondratska K, Skryma R, Slomianny C, Prevarskaya N (2013). Calcium-permeable ion channels in control of autophagy and cancer. Front Physiol.

[47] Corazao-Rozas P, Guerreschi P, Andre F, Gabert PE, Lancel S, Dekiouk S, Fontaine D, Tardivel M, Savina A, Quesnel B (2016). Mitochondrial oxidative phosphorylation controls cancer cell’s life and death decisions upon exposure to MAPK inhibitors. Oncotarget..

[48] Cunningham JT, Rodgers JT, Arlow DH, Vazquez F, Mootha VK, Puigserver P (2007). mTOR controls mitochondrial oxidative function through a YY1-PGC-1alpha transcriptional complex. Nature..

[49] Duval C, Chinetti G, Trottein F, Fruchart JC, Staels B (2002). The role of PPARs in atherosclerosis. Trends Mol Med.

[50] Jarvis MC, Gray TJ, Palmer CN (2005). Both PPARgamma and PPARdelta influence sulindac sulfide-mediated p21WAF1/CIP1 upregulation in a human prostate epithelial cell line. Oncogene..

[51] Lo SH, Lee KS, Chen LJ, Cheng JT, Chen CH (2013). Increase of PPARdelta by dopamine mediated via DA-1 receptor-linked phospholipase C pathway in neonatal rat cardiomyocytes. Auton Neurosci.

[52] Suh HN, Huong HT, Song CH, Lee JH, Han HJ (2008). Linoleic acid stimulates gluconeogenesis via Ca2+/PLC, cPLA2, and PPAR pathways through GPR40 in primary cultured chicken hepatocytes. Am J Physiol Cell Physiol.

