# Supplementary material for: PLCε regulates prostate cancer mitochondrial oxidative metabolism and migration via upregulation of Twist1
Source: J Exp Clin Cancer Res. 2019 Aug 5;38:337. doi: 10.1186/s13046-019-1323-8 (PMC6683382; doi:10.1186/s13046-019-1323-8)
Supplement: Supplementary file 1 — Figure S1. High expression levels of PLCε and Twist1 in PCa tissues. (a) RNA-seq mRNA expression data from the TCGA and GTEx database was used to compare Twist1 expression between in PCa tumors (T) (n = 492) and their non-tumor counterparts (N) (n = 152). *p < 0.05. (b) Correlation between the expression of Twist1 and PLCε in PCa patients from cBioPortal database. Figure S2. PLCε expression negatively correlates with PGC-1α-mediated mitochondrial oxidative metabolism and uncoupling through Twist1 in DU145 and Bica-R cells. Figure S3. PLCε depletion decreases phosphorylation of Twist1 on serine 68 and reduces stability of Twist1 protein via MAPKs in DU145 and Bica-R cells. Figure S4 PLCε depletion decreases Twist1 protein via MAPKs and alleviates the PPARβ expression. Figure S5 PLCε depletion decreases Twist1 by PPARβ. (DOCX 3776 kb) [file 13046_2019_1323_MOESM1_ESM.docx]

**Additional file 1**

**Figure titles and legends**

Figure S1 is related to Figure 1.

Figure S2 is related to Figure 4.

Figure S3 is related to Figure 5.

Figure S4 is related to Figures 5 and 6

Figure S5 is related to Figure 7

**Figure S1** High expression levels of PLCε and Twist1 in PCa tissues. (a) RNA-seq mRNA expression data from the TCGA and GTEx database was used to compare Twist1 expression between in PCa tumors (T) (n = 492) and their non-tumor counterparts (N) (n = 152). * p < 0.05. (b) Correlation between the expression of Twist1 and PLCε in PCa patients from cBioPortal database.

**Figure S2** PLCε expression negatively correlates with PGC-1α-mediated mitochondrial oxidative metabolism and uncoupling through Twist1 in DU145 and Bica-R cells. (a, e and i) Western blotting analysis detected the expression of PLCε, Twist1, PGC-1α, CPT1B, ERRα, UCP-1, and ACADM after infected with lentiviral sh-PLCε or transfected with DDK-Twist1 or sh-Twist1 plasmids. (b, f and j) Proteins were quantified using image software normalized against β-actin in DU145 and Bica-R cells. (c, g and k) Seahorse tracing of the oxygen consumption rate in DU145 and Bica-R cells, followed by mitochondrial stress test as described in methods. (d, h and l ,f) Bar graphs of means ± SD of the basal and maximal respiration and ATP production in DU145 and Bica-R cells. Data were represented as mean ± SD. of three individual experiments. *p< 0.05, **p< 0.01, and *** p<0.001 vs. Controls.

**Figure S3** PLCε depletion decreases phosphorylation of Twist1 on serine 68 and reduces stability of Twist1 protein via MAPKs. (a, b) Western blotting and protein quantification analyses detected the expression of B-Raf, p-MEK, t-MEK, p-ERK, t-ERK, MEKK3, MEK4, p-P38, P38, MEKK2, p-JNK and t-JNK after knockdown PLCε in DU145 and Bica-R cells. (c) Western blotting and protein quantification analyses detected the expression of p-ERK in different concentration of trametinib. (d) Protein expression of p-JNK in different concentration of JNK-In-8. (e) Protein expression of p-P38 in different concentration of SB203580. (f, g) Western blotting and protein quantification analyses detected the expression of PLCε, Twist1, pSer68, B-Raf, p-MEK, t-MEK, p-ERK and t-ERK in DU145 and Bica-R cells treated with 1 nM trametinib. (h, k) Q-PCR analysis of PLCε and Twist1 in DU145 and Bica-R cells treated with trametinib or JNK-In-8. (i, j) Western blotting and protein quantification analyses detected the expression of PLCε, Twist1, pSer68, MEKK2, p-JNK and t-JNK in DU145 and Bica-R cells treated with 5 nM JNK-In-8. Data were represented as mean ± SD. of three individual experiments. *p< 0.05, **p< 0.01, and *** p<0.001 vs. Controls.

**Figure S4** PLCε depletion decreases Twist1 protein via MAPKs and alleviates the PPARβ expression. (a,b) Western blotting and protein quantification analyses detected the expression of PLCε, Twist1, pSer68, MEKK3, MEK4, p-P38 and P38 in PCa cells treated with SB203580. (c) Q-PCR analysis of PLCε and Twist1 in PCa cells treated with SB203580. (d, e) Western blotting and protein quantification analyses detected the expression of PLCε, PPARβ, PPARα and PPARγ in sh-PLCε-infected DU145 and Bica-R cells. (f) The protein quantification analyses the expression of PLCε and PPARβ after knockdown of PPARβ in PC3 cells line. Data were represented as mean ± SD. of three individual experiments. *p< 0.05, **p< 0.01, and *** p<0.001 vs. Controls.

**Figure S5** PLCε depletion decreases Twist1 by PPARβ. (a, b) Treatment of sh-PLCε-transfected DU145 and Bica-R cells with 1 nM GW501516 and 1 µM GSK3787, cell lysates were collected for western blotting and protein quantification analyses. (c) qRT-PCR analysis of PLCε, PPARβ and Twist1 mRNA expression in PCa cells. (d, e) Western blotting and protein quantification analyses detected the expression of PPARβ and Twist1 PC3 cells after PLCε or . PPARβ knocked down. (f, g) Protein lysates from sh-PLCε-infected DU145 cells treated with GW501516 and GSK3787 were collected for western blotting and protein quantification analyses. Data were represented as mean ± SD. of three individual experiments. *p< 0.05, **p< 0.01, and *** p<0.001 vs. Controls.

**Figure S1**


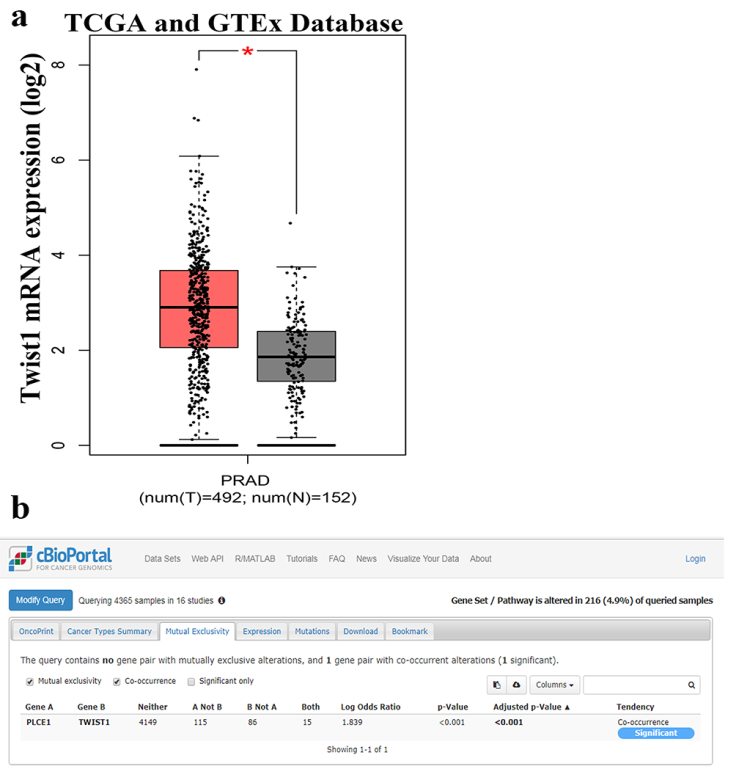


**Figure S2**

**
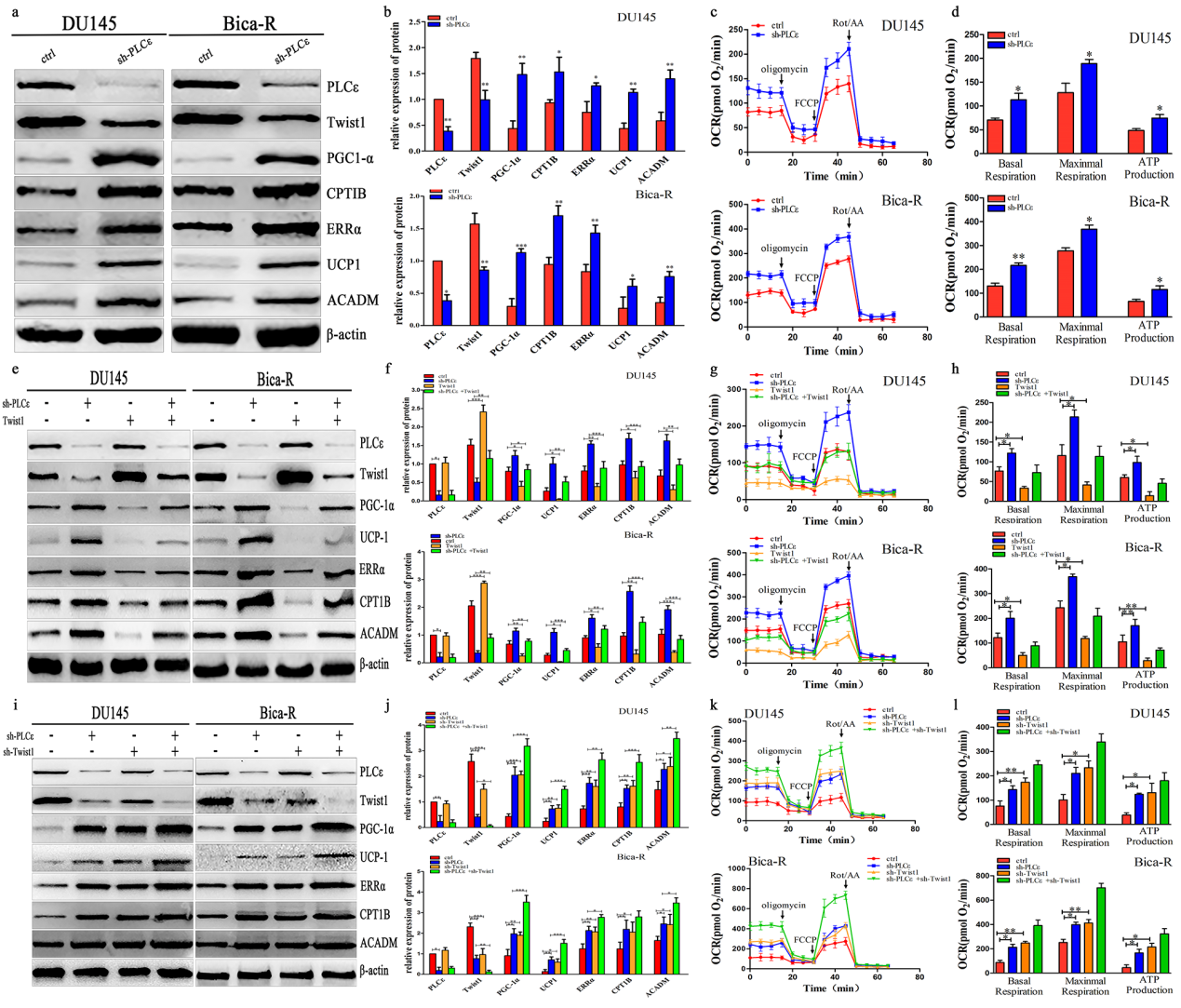
**

**Figure S3**


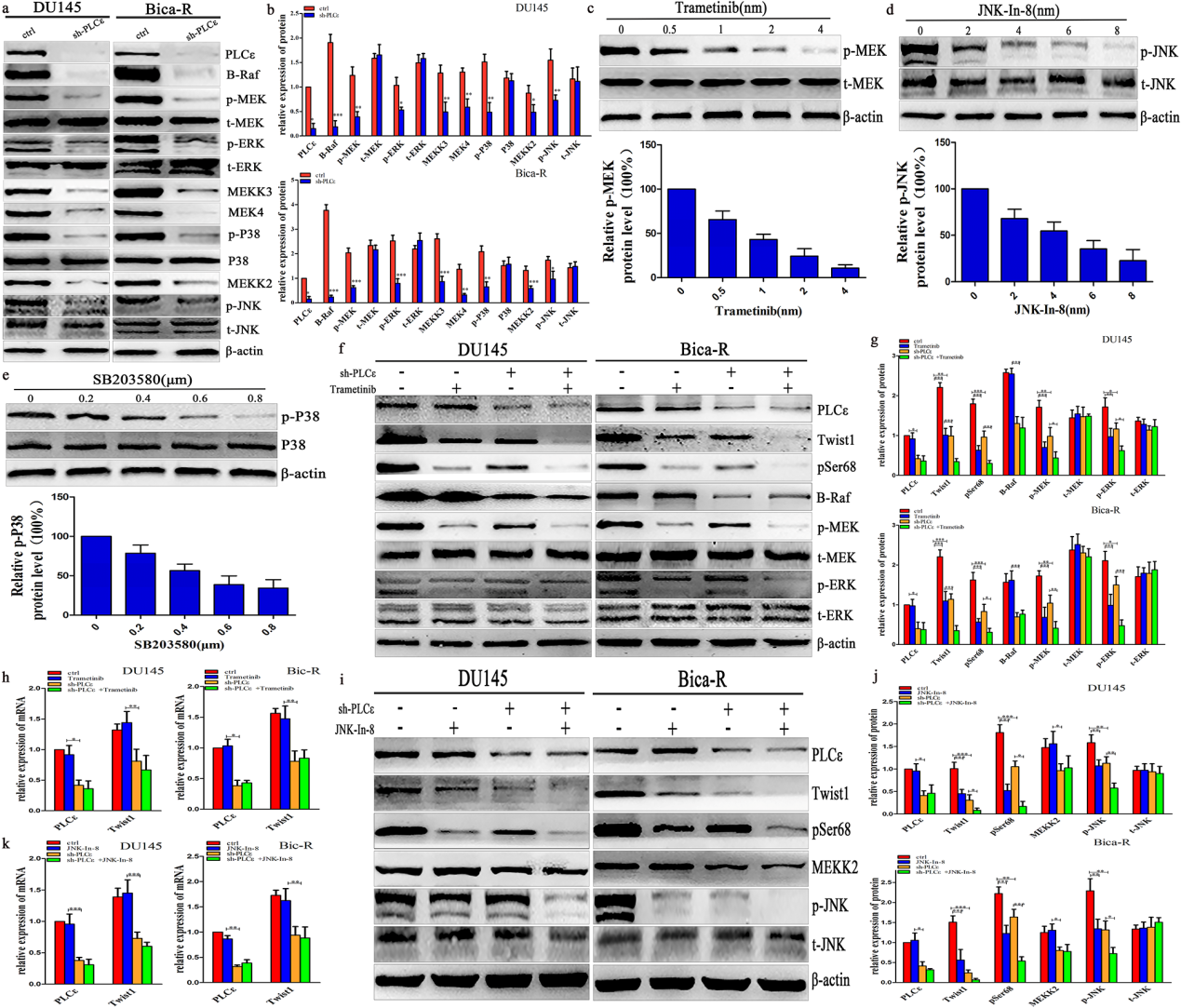


**Figure S4**

**
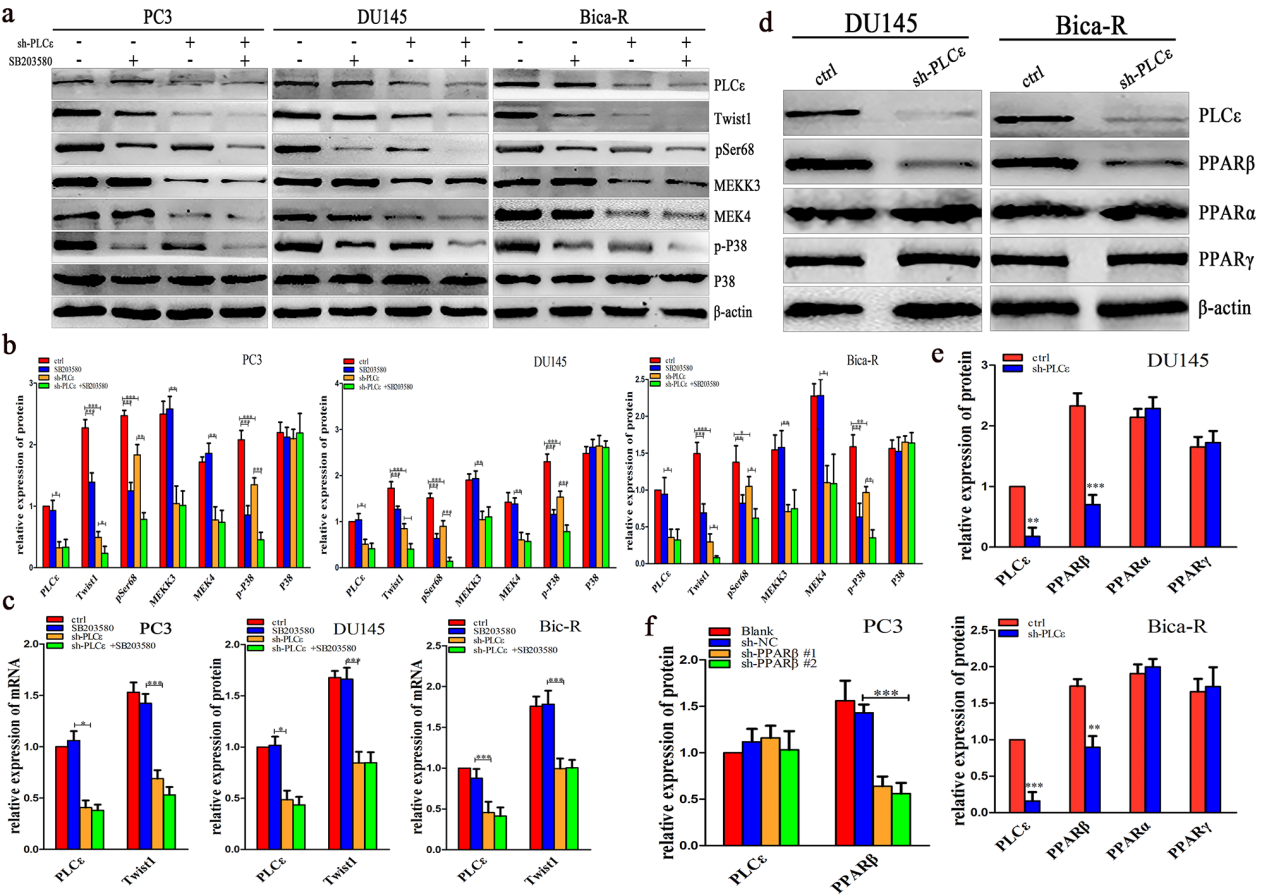
**

**Figure S5**

**
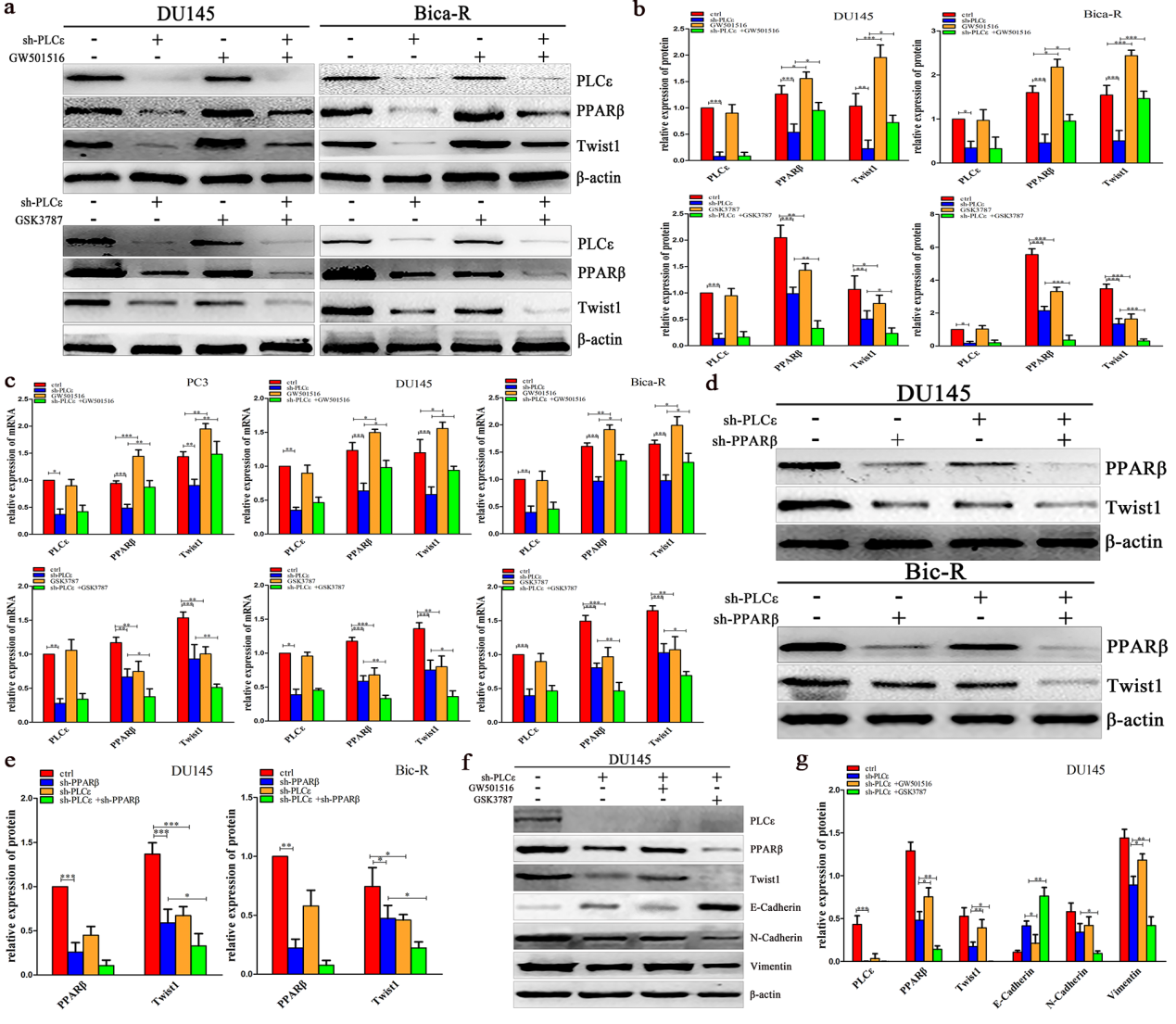
**
